# Thickness-dependent polaron crossover in tellurene

**DOI:** 10.1126/sciadv.ads4763

**Published:** 2025-01-08

**Authors:** Kunyan Zhang, Chuliang Fu, Shelly Kelly, Liangbo Liang, Seoung-Hun Kang, Jing Jiang, Ruifang Zhang, Yixiu Wang, Gang Wan, Phum Siriviboon, Mina Yoon, Peide D. Ye, Wenzhuo Wu, Mingda Li, Shengxi Huang

**Affiliations:** ^1^ Molecular Biophysics and Integrated Bioimaging Division, Lawrence Berkeley National Laboratory, Berkeley, CA 94720, USA.; ^2^ Department of Chemistry, University of California, Berkeley, CA 94720, USA.; ^3^ Department of Nuclear Science and Engineering, Massachusetts Institute of Technology, Cambridge, MA 02139, USA.; ^4^ X-ray Science Division, Argonne National Laboratory, Lemont, IL 60439, USA.; ^5^ Center for Nanophase Materials Sciences, Oak Ridge National Laboratory, Oak Ridge, TN 37831, USA.; ^6^ Materials Science and Technology Division, Oak Ridge National Laboratory, Oak Ridge, TN 37831, USA.; ^7^ Edwardson School of Industrial Engineering, Purdue University, West Lafayette, IN 47907, USA.; ^8^ Department of Mechanical Engineering, Stanford University, Stanford, CA 94305, USA.; ^9^ Department of Physics, Massachusetts Institute of Technology, Cambridge, MA 02139, USA.; ^10^ Elmore Family School of Electrical and Computer Engineering, Purdue University, West Lafayette, IN 47907, USA.; ^11^ Purdue Quantum Science and Engineering Institute, Purdue University, West Lafayette, IN 47907, USA.; ^12^ Department of Electrical and Computer Engineering and the Rice Advanced Materials Institute, Rice University, Houston, TX 77005, USA.

## Abstract

Polarons, quasiparticles from electron-phonon coupling, are crucial for material properties including high-temperature superconductivity and colossal magnetoresistance. However, scarce studies have investigated polaron formation in low-dimensional materials with phonon polarity and electronic structure transitions. In this work, we studied polarons of tellurene, composed of chiral Te chains. The frequency and linewidth of the A_1_ phonon, which becomes increasingly polar for thinner tellurene, change abruptly for thickness below 10 nanometers, where field-effect mobility drops rapidly. These phonon and transport signatures, combined with phonon polarity and band structure, suggest a crossover from large polarons in bulk tellurium to small polarons in few-layer tellurene. Effective field theory considering phonon renormalization in the small-polaron regime semiquantitatively reproduces the phonon hardening and broadening effects. This polaron crossover stems from the quasi–one-dimensional nature of tellurene, where modulation of interchain distance reduces dielectric screening and promotes electron-phonon coupling. Our work provides valuable insights into the influence of polarons on phononic, electronic, and structural properties in low-dimensional materials.

## Teaser

Polaron crossover in ultrathin tellurene markedly alters its phonon behavior and electronic transport properties.

## INTRODUCTION

Polaron is a quasiparticle formed by the coupling between charge carriers and lattice vibrations (phonons). The interaction between charge carriers and phonons induces a polarization cloud that follows the charge carriers, which can lead to higher effective mass, lower mobility, and hopping-like conductivity, distinct from nearly free electrons (*1*–*4*). Because of its unique features, polaron plays a crucial role in various physical properties, such as electronic transport, magnetoresistance, ferroelectricity, and thermoelectricity (*5*, *6*). For example, the strong Jahn-Teller effect in La_1−*x*_Ca*_x_*MnO_3+*y*_ can lead to the formation of polarons that is associated with the high-temperature superconductivity (*7*–*9*). Depending on the coupling strength between electrons and phonons, polarons can be categorized into small polarons and large polarons on the basis of the spatial distribution (*5*). For small polarons with small spatial extension, the electron-phonon coupling is strong compared to the kinetic energy of electrons. In this strong coupling regime, the electron becomes localized within a regime of a size comparable to the unit cell, forming small polarons (*3*, *10*). In contrast, large polarons with large-polaron radii are formed in the weak coupling regime where the electron can delocalize over a larger region in the lattice (*11*). In solid-state materials, the crossover from large polarons to small polarons (weak coupling to strong coupling) is often associated with metal-to-semiconductor/insulator transitions, with scattered reports insofar (*12*, *13*). In La_1−*x*_Ca*_x_*MnO_3_, the transition from large polaron at low temperature to small polaron at high temperature can be revealed by the characteristic Raman scattering response at metal-to-semiconductor transition (*13*). This polaron crossover has also been quantitatively measured by the Jahn-Teller polaronic distortion using extended x-ray absorption fine structure (EXAFS) analysis where the Mo─O bond length showed noticeable changes (*12*).

Tellurene serves as an ideal platform to study polarons because of the relatively large polaron coupling constant (*14*). Tellurene is composed of helical chains of tellurium atoms and hosts unique properties that make it promising for applications such as broadband photodetectors (*15*, *16*), high-mobility field-effect transistors (FET) (*17*), topological phase change transistors (*18*), and valley transistors (*19*). The carrier mobility of tellurene FET shows a dependence on the film thickness, consistent with the increase of bandgap from bulk tellurium to few-layer tellurene (*17*). The dependence of electronic structure on thickness also leads to different topological states at varied temperatures. In topological phase change transistors based on tellurene, thick tellurene of 32 nm exhibits negative magnetoresistance below 100 K, a signature of topological states, while thin tellurene of 12 nm shows only positive magnetoresistance (*18*). These transport behaviors modulated by tellurene thickness suggest that the polaronic properties of tellurene can strongly depend on its thickness.

In this work, we investigate the polarons of tellurene and the crossover from large polaron to small polaron as tellurene thickness reduces. In thinner tellurene, the longitudinal A_1_ phonon becomes polar as opposed to nonpolar in bulk tellurium, as shown by density functional theory (DFT) calculations. This distinct increase in phonon polarity, a requirement for forming small polarons, strongly indicates the presence of small-polaron/large-polaron crossover dependent on tellurene thickness. The transition from large polarons in bulk tellurium to small polarons in few-layer tellurene is also exhibited in our measured A_1_ phonon, which shows a blue shift by 10 cm^−1^ and a broadening by 5 cm^−1^ for tellurene thickness below 10 nm. To understand the influence of polarons on phonon properties, we developed a theoretical model to explain the phonon hardening and broadening effect due to polaron formation. Specifically, the small polaron contributes to strong renormalization of phonon frequency and linewidth, which is absent in the large-polaron regime. This polaron crossover as a function of tellurene thickness can be understood by the structural transition of tellurene as its thickness reduces. EXAFS spectroscopy reveals that the distance between neighboring Te chains changes drastically for smaller thicknesses, which is in agreement with the formation of small polarons due to reduced dielectric screening and enhanced electron-phonon coupling. Our unified experimental, computational, and theoretical study provides a comprehensive picture of the thickness-dependent polaron formations in few-layer tellurene. The understanding of polaron formation and its implication for electronic structure and phonon response is fundamental for tailoring the electronic properties of tellurene-based devices.

## RESULTS

### Thickness dependence of Raman signatures in few-layer tellurene

**Fig. 1.**
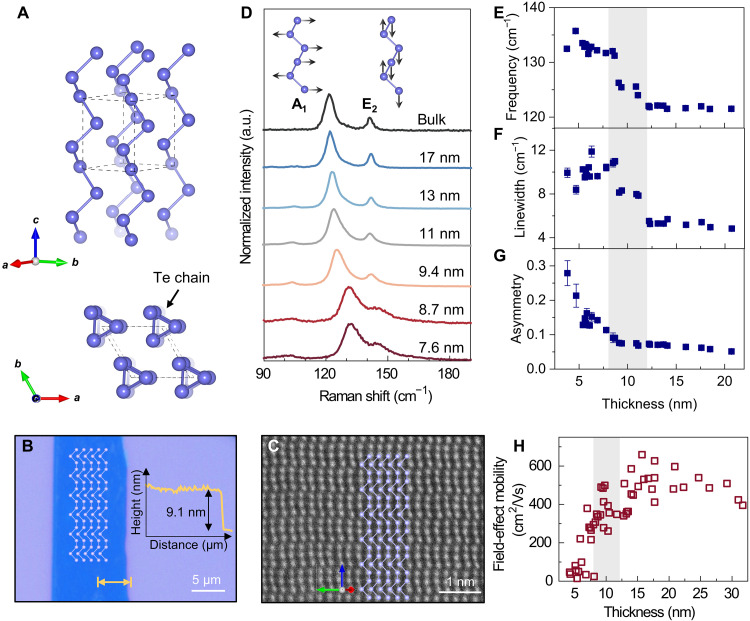
Thickness-dependent structural, phononic, and electronic properties of tellurene. (**A**) The crystal structure of tellurene. (**B**) Optical image of tellurene with a thickness of 9.1 nm. Inset: Depth profile across the arrow. (**C**) Scanning transmission electron microscopy images of tellurene. (**D**) Raman spectra for tellurene with different thicknesses. Inset: Lattice vibration of the A_1_ and E_2_ phonon modes. (**E** to **G**) The A_1_ phonon frequency, linewidth, and asymmetry as a function of thickness. (**H**) The field-effect mobility of tellurene as a function of thickness. (H) Adapted from (*17*).

Tellurene is the two-dimensional (2D) form of bulk tellurium, which is composed of chiral chains of tellurium atoms in a hexagonal lattice as shown in Fig. 1 (A and C). Each tellurium atom is covalently bonded to the two nearest atoms within the same chain, while the neighboring chains interact through weak van der Waals force (*20*). The few-layer tellurene synthesized by the hydrothermal method (*17*) has a typical thickness of a few nanometers and a lateral dimension in micrometers as measured by atomic force microscopy (Fig. 1B and fig. S1). As shown in Fig. 1D, bulk tellurium exhibits the A_1_ mode at 121 cm^−1^ and the E_2_ mode at 141 cm^−1^. These phonon modes correspond to the chain expansion in the basal plane and asymmetric stretching along the *c* axis, respectively. The polarization-dependent measurement in fig. S2 reveals the phonon symmetry, which is consistent with prior reports on α-phase tellurium (*21*). Because the A_1_ mode exhibits asymmetry in the spectral line shape, we performed the fitting using the Breit-Wigner-Fano (BWF) function rather than symmetric Lorentzian or Gaussian functions (note S1 and fig. S3). The BWF function is written as $I\left(\omega \right)=I_{0}\frac{{\left[1+\left(\omega -\omega_{0}\right)/\left(q_{s}\Gamma_{0}/2\right)\right]}^{2}}{1+{\left[\left(\omega -\omega_{0}\right)/\left(\Gamma_{0}/2\right)\right]}^{2}}$, where $I_{0}$, $\omega_{0}$, and $\Gamma_{0}$ denote the intensity, frequency, and linewidth of the phonon mode, respectively. 1/*q*_s_ represents the asymmetry of the spectral line shape that deviates from the standard Lorentzian line shape. When 1/*q*_s_ = 0, the spectrum returns to symmetric Lorentzian line shape.

As the thickness of tellurene reduces, the A_1_ mode exhibits a blue shift of more than 10 cm^−1^ accompanied by a broadening of the linewidth (Fig. 1D), in agreement with publications of few-layer tellurene (*22*–*24*). The fitting results of the A_1_ phonon as a function of tellurene thickness are summarized in Fig. 1 (E to G). For tellurene with a thickness from 20 to 5 nm, the frequency of the A_1_ phonon increases from 121 to 133 cm^−1^ (Fig. 1E). The overall increase of A_1_ phonon frequency can be partly understood by the increased intrachain bond strength for thinner layers, which contributes to a more effective restoring force for the chain expansion of the A_1_ mode. However, this cannot explain the drastic transition of phonon frequency at the thickness of 10 ± 2 nm (Fig. 1E). This is consistent with prior reports of phonon frequencies of tellurene synthesized using the same solution-phase hydrothermal approach (*17*). Furthermore, a similar drastic phonon transition has been observed in 2D tellurene synthesized through topotactic transformation from MoTe_2_ to Te (*25*). Although previous studies on thickness-dependent Raman scattering of tellurium have not fully explained the underlying physics, the consistent observations across different synthesis methods suggest that this frequency shift is an intrinsic property of tellurene. At the same critical thickness, the linewidth of the A_1_ mode also shows considerable increases approximately from 2.5 to 5 cm^−1^ (Fig. 1F), suggesting a change in the phonon lifetime. In addition, the asymmetry parameter 1/*q*_s_ becomes proportional to the thickness below 10 nm, with a maximum 1/*q*_s_ = 0.28 for a 4-nm tellurene as shown in Fig. 1G.

The asymmetric line shape of the A_1_ phonon mode suggests the presence of Fano resonance in tellurene with varied thicknesses. Fano resonance is a phenomenon arising from the interaction between the scattering of a discrete mode with the scattering of an electronic continuum (*26*). This interaction modifies the discrete phonon, leading to changes in the line shape and broadening of the linewidth. The 1/*q*_s_ factor not only describes the asymmetry of the spectrum but also is related to the interaction strength between the discrete and continuous states. Such Fano resonance has been widely observed in phonon spectra of quantum materials such as Weyl semimetals (*27*–*29*) and Dirac semimetals (*30*–*32*). In our case of tellurene, the observed Fano resonance is attributed to electron-phonon coupling due to the interaction between the A_1_ phonon and the valence electrons. In particular, the drastic increase of 1/*q*_s_ below 10 nm suggests an increase in electron-phonon coupling. These characteristic phonon features can be understood by the formation of small polarons in tellurene as a result of loss of dielectric screening below the critical thickness. As the thickness of tellurene reduces, the weaker dielectric screening leads to enhanced electron-phonon coupling, contributing to a stronger Fano resonance and an increased asymmetry of the A_1_ mode (Fig. 1G).

Moreover, the formation of small polarons raises the electrical resistance due to strong electron-phonon interaction to localize the electrons. This is consistent with the changes in field-effect mobility of tellurene as shown in Fig. 1H, which experiences an immediate decrease around 10 ± 2 nm from 600 cm^2^ V^−1^ s^−1^ down to 50 cm^2^ V^−1^ s^−1^ (*17*). In addition, few-layer tellurene also exhibits thickness-dependent thermal conductivity with a nonmonotonic relationship with Te thickness. A sharp transition occurs at 10 nm, attributed to the onset of 2D phonon transport, which deviates from 3D phonon transport in bulk (*33*, *34*). In particular, the nonmonotonic behavior further hints at the contribution beyond only 2D-to-3D dimension crossover and opens the possibility for small-polaron formation. Similar to the electronic behavior, this critical thickness of thermal conductivity again aligns with what we observed for the A_1_ optical phonons. It has been reported that strong scattering between optical phonons and acoustic phonons can affect the thermal conductivity of 2D tellurene (*35*). Therefore, it is likely that the unique transition of thermal conductivity is also linked to optical phonons and their interaction with charge carriers.

### Thickness-dependent phonon polarity and bandgap: A first-principles study

**Fig. 2.**
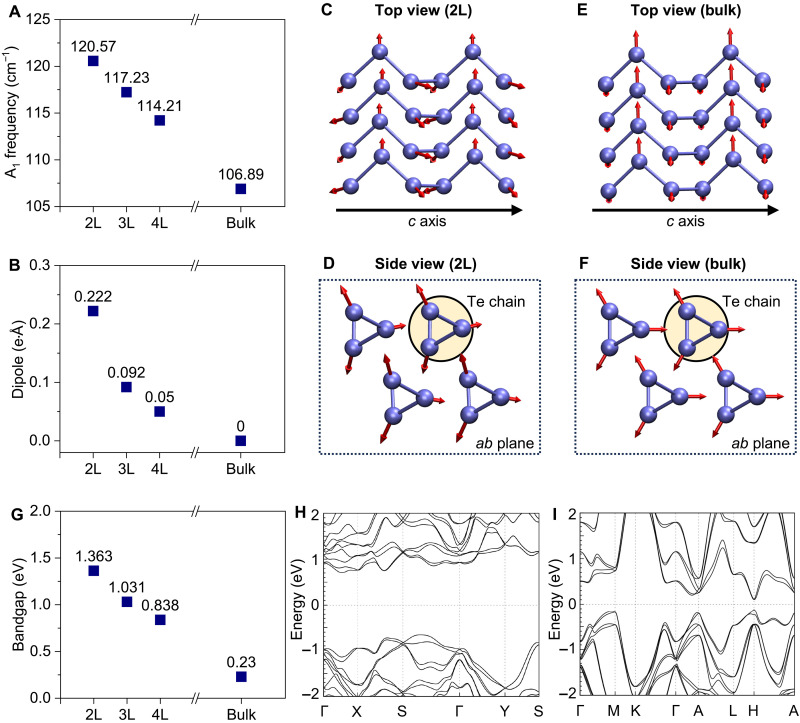
Calculated phonon polarity and band structure for few-layer tellurene and bulk tellurium. (**A**) The calculated A_1_ phonon frequency. (**B**) The calculated change of the dipole moment by the A_1_ mode as a function of thickness. (**C** to **F**) Top view and side view with respect to the experiment geometry showing the calculated lattice vibrations of the A_1_ mode in 2L tellurene and bulk tellurium. The red arrows represent the atomic vibrations. (**G**) The calculated bandgap of tellurene as a function of thickness. Calculated band structure of (**H**) 2L tellurene and (**I**) bulk tellurium.

To understand the change of the A_1_ phonon, we performed first-principles calculations of the phonon properties and electronic structure. The overall blue shift of the phonon modes in few-layer tellurene as compared to bulk tellurium can be reproduced by calculations (Fig. 2A and fig. S4). According to a prior experimental study (*14*), among the four optical phonon modes at the gamma point in bulk Te, only the A_1_ phonon is nonpolar and does not change the electric dipole moment (or polarization) of the system. This is confirmed by our calculations where the change of dipole moment caused by the A_1_ phonon is zero in bulk tellurium (Fig. 2B). However, our calculations find out that the A_1_ phonon becomes increasingly polar with a decreasing thickness, as shown in Fig. 2B, where the change of the dipole moment caused by the A_1_ phonon becomes drastically larger in few-layer tellurene as compared to bulk tellurium. As the A_1_ mode vibrates in the *ab* plane, which is perpendicular to the chiral chain direction along the *c* axis (Fig. 1A), the change of the dipole moment is also perpendicular to the chain. In bulk tellurium, the contributions of Te atoms to the change of dipole moment by the A_1_ phonon perfectly cancel out one another as illustrated in Fig. 2 (E and F) (vibrations of different Te atoms are symmetrical to one another). With a decreased number of layers, the exterior Te atoms become increasingly nonequivalent to the interior Te atoms (*36*); therefore, their contributions to the change of dipole moment can no longer be canceled out by the interior Te atoms. This can be seen from the nonsymmetrical vibrations of Te atoms in 2L tellurene (Fig. 2, C and D). Our calculations suggest a nonpolar to polar transition that occurs at a certain thickness for the A_1_ mode, in contrast to the other three optical phonons (E_1_, E_2_, and A_2_) that are always polar irrespective of the thickness (Fig. 2B). Such difference could contribute to the unique thickness dependence of the A_1_ mode, especially the marked change of its frequency and linewidth at a certain sample thickness.

In addition, the calculated band structure shows the transition from semimetal to semiconductor, where the calculated bandgap of 0.23 eV of bulk tellurium increases to 1.363 eV in bilayer tellurene (Fig. 2, G to I, and fig. S5), leading to a thickness-dependent optical absorption (fig. S6). The corroborated evolutions of phonon property and electronic structure point to the formation of small polarons in few-layer tellurene, where the localized small polarons cause the renormalization of phonon frequency and linewidth as compared to large polarons in bulk tellurium.

### Polaron theory for phonon renormalization

To describe the effect of polaron formation on phonon dispersion and linewidth, we start with a Hamiltonian consisting of three parts (*37*)

$$H=-t\sum_{\left\langle \mathrm{ij}\right\rangle }c_{i}^{†}c_{j}+\sum_{q}\omega_{q}a_{q}^{†}a_{q}+\sum_{jq}g_{q}c_{j}^{†}c_{j}\left(a_{q}+a_{-q}^{†}\right)e^{iq\cdot R_{j}}$$ (1)

**Fig. 3.**
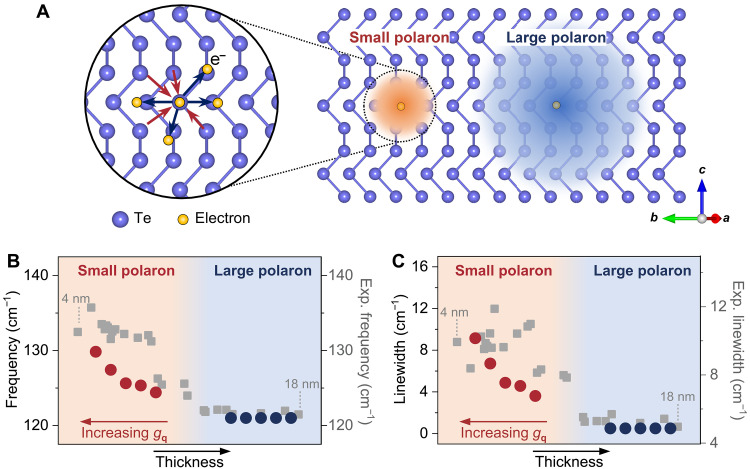
Theory-predicted phonon hardening and broadening effect. (**A**) Illustration of polarons. Left: Arrows represent attractive (red) and repulsive (blue) forces. Right: The red sphere with a small radius and the blue sphere with a large radius represent the formation of the small polaron and large polaron, respectively. (**B**) The theoretically predicted phonon frequency for small polaron and large polaron. The thickness dependence is schematically sketched. (**C**) The theoretically predicted one-loop polaron corrected phonon linewidth for small polaron and large polaron. The theoretical linewidth only includes the polaron contribution. The thickness dependence is schematically sketched. The experimental data for tellurene from 4 to 18 nm are shown by the closed squares.

The first two terms represent the noninteracting electrons and noninteracting phonons where t is the electron hopping, $\omega_{q}$ is the phonon energy at wave vector **q**, and we have normalized ℏ to be 1. Here, we use $c_{i}^{†}\left(c_{i}\right)$, $a_{q}^{†}\left(a_{q}\right)$ to denote the creation (annihilation) operator of electron at site *i* and phonon at momentum **q**, respectively. The third term denotes the electron-phonon coupling with $g_{q}$ representing the coupling strength. This Hamiltonian can describe the behavior of polarons, as detailed in (*37*). Depending on the strength of electron-phonon coupling, polarons can be classified into small polarons (with strong electron-phonon coupling) and large polarons (with weak electron-phonon coupling) as illustrated in Fig. 3A. In our case, the thinner Te sample with polar phonon and strong electron-phonon coupling prefers to form small polarons while the thicker Te sample forms large polarons. For the large polaron within the weak coupling regime, the change of the phonon frequency and linewidth is derived at the order of magnitude proportional to ${|g_{q}|}^{2}$. Because $g_{q}$ is relatively small in the weak coupling regime for large polarons, the phonon frequency blue shift should be small, and the phonon linewidth is narrower for thicker tellurene. Therefore, theoretically, large polarons do not strongly affect phonon properties, whereas small polarons can influence both phonon frequency and linewidth (details in note S2).

For the small polaron within the strong coupling regime, the Hamiltonian can be rewritten using the Lang-Firsov canonical transformation (*37*) where the electron operator is transformed into the polaron operator

$$H=-\sum_{j}E_{B}c_{j}^{+}c_{j}+\sum_{q}\omega_{q}a_{q}^{+}a_{q}-t\sum_{\left\langle \mathrm{ij}\right\rangle }\text{exp}\left(-\sum_{q}T_{q\mathrm{ij}}p_{q}e^{-iq\cdot R_{i}}\right)c_{i}^{+}c_{j}$$ (2)

where $T_{q\mathrm{ij}}=\frac{g_{q}^{*}}{\omega_{q}}i\sqrt{\frac{2}{m\omega_{q}}}\left[1-e^{iq\cdot \left(R_{i}-R_{j}\right)}\right]$ represents the lattice distortion due to the polaron, and $p_{q}=i\sqrt{\frac{m\omega_{q}}{2}}\left(a_{q}^{+}-a_{-q}\right)$ is the momentum operator of the phonon, $E_{B}=\sum_{q}\frac{{|g_{q}|}^{2}}{\omega_{q}}$ is the normalized small-polaron binding energy. Here, we can separate the lattice distortion into the effective tight-binding model, phonon, and the phonon-polaron interaction Hamiltonian.

With the help of the equation of motion method for the phonon’s Green’s function (*38*), the theory indicates the phonon frequency dressed by the small polaron will be renormalized

$$\Omega_{q}=\omega_{q}\left[1+\frac{4{|g_{q}|}^{2}}{\omega_{q}^{2}N}\sum_{\mathrm{kn}}\frac{te^{-S_{n}}}{\omega_{q}}\left(1-\text{cos}q\cdot R_{n}\right)n_{Fk}e^{+ik\cdot R_{n}}\right]$$ (3)

where $S_{n}=\frac{2}{N}\sum_{q}\frac{{|g_{q}|}^{2}}{\omega_{q}^{2}}{\text{sin}}^{2}\left(\frac{q\cdot R_{n}}{2}\right)\left(2n_{Bq}+1\right)$. The $e^{-S_{n}}$ term is the effective factor of electron hopping, which reflects the localization of the small polaron and leads to the reduction of carrier mobility. $n_{Fk}$ and $n_{Bq}$ are the corresponding Fermi-Dirac distribution and Bose-Einstein distribution, which introduce the temperature dependence of the normalized frequency. For polar phonon (which is shown to only exist in thin tellurene), the electron-phonon coupling can develop a long-range interaction, i.e., $g_{q}^{*}\propto \frac{1}{q}$, resulting in a finite polaron-phonon coupling when ${|g_{q}|}^{2}$ and $\left(1-\text{cos}q\cdot R_{n}\right)$ cancel the q dependence at the q→0 limit, and then $\Omega_{q}$ should have an enhancement with strong electron-phonon coupling. For semimetallic bulk tellurium, dielectric screening only results in weak electron-phonon coupling. Hence, no phonon hardening effect is observed for thick tellurene films corresponding to large polarons (Fig. 3B, blue dots). Whereas few-layer tellurene is semiconducting, the reduced dielectric screening can lead to long-range interactions between polar A_1_ phonons and electron clouds, contributing to the phonon hardening effect for small polarons (Fig. 3B, red dots). This is consistent with the experimental observation where the phonon frequency is increased drastically by 12 cm^−1^ as the thickness decreases below 10 nm (Fig. 1B). The phonon frequency also exhibits different temperature dependencies when comparing small polarons and large polarons (figs. S7 and S8). Figure S8 plots the frequency difference of the A_1_ phonon between thin and thick tellurene (with thickness below and above the polaron transition). In the experiment, the frequency difference continues to decrease at lower temperatures, which aligns with our theoretical predictions of the frequency difference between small and large polarons.

To obtain the phonon linewidth, we perform the perturbation of the hopping amplitude and take the one-loop polaron “polarization operator” correction as an approximation to get the self-energy of a phonon; the phonon linewidth from the small polaron can be derived as (note S2)

$$\Gamma_{q}=\frac{\omega_{q}}{\Omega_{q}}\delta \left(\omega_{q}-\Delta \right)\frac{2\pi e^{2}t^{2}}{\omega_{q}}\left(\frac{1}{\varepsilon_{\infty }}-\frac{1}{\varepsilon_{0}}\right)\sum_{n}\frac{{\left(q\cdot R_{n}\right)}^{2}}{q^{2}}$$ (4)

where $\Delta \approx \underset{q\to 0}{\text{lim}}\varepsilon_{p+qb}-\varepsilon_{pa}$ is the gap of the polaron band, and *a* and *b* are band indices as we require multiple polaron bands to describe the semiconducting feature of the few-layer tellurene under the quasiparticle picture. For simplicity, we make use of the two-band model by assuming the initial polaron state is fully occupied, $n_{F}\left(\varepsilon_{pa}\right)=1$, and the final state is empty, $n_{F}\left(\varepsilon_{p+qb}\right)=0$, and the phonon energy is around Δ to assist the interband transition from the initial state to the final state. $\varepsilon_{0}$ and $\varepsilon_{\infty }$ are the related dielectric constant. Then, the phonon linewidth will exhibit a finite increase due to the influence of small polaron compared to the case of large polaron when q→0. As we experimentally observe the increase of linewidth when Te thickness is reduced (Fig. 1E), this increase is also captured from our theory through the one-loop polaron correction perturbation theory estimation (Fig. 3C). The discrepancy between the absolute linewidths from the experiment and theory can be attributed to the fact that our polaron theory only considers the linewidth broadening due to electron-phonon interaction, while other factors, such as phonon-phonon interaction and defect scattering, can also affect the measured phonon linewidth. Overall, the agreement between the experiment and theory indicates that the enhanced electron-phonon scattering dominates the increase of linewidth for few-layer tellurene. It is also worthwhile mentioning that because small-polaron and large-polaron theory begin with different canonical bases, a unified theory that treats both, including predicting crossover directly, has not been developed to the best of our knowledge. Nevertheless, our DFT calculation shows strong evidence of the polar A_1_ phonon, which provides the basis of our picture; therefore, we computed the phonon properties based on small polaron for thinner films and large-polaron for thicker films, separately.

As a result, our theory can well explain the phonon behaviors observed in few-layer tellurene by phonon renormalization in the small-polaron (strong coupling) regime on a semiquantitative level. All the numerical calculations based on the derived polaron-phonon theory give a minimal estimation with a clear physical picture and align with the experimental observation in Fig. 3. Besides phonon behaviors, the observed sudden drop of carrier mobility (Fig. 1H) due to the formation of small polaron can also be interpreted from a theoretical viewpoint, as the electron-phonon coupling evidenced by the increase of the phonon linewidth will lead to the drop of carrier mobility (note S3). More specifically, the developed small polaron is spatially localized because of the strong electron-phonon scattering. As a result, the effective hopping, which dominates the carrier mobility, is reduced. This further confirms the polaron crossover from large polaron (weak coupling regime) in the bulk tellurium to small polaron (strong coupling regime) in a few-layer tellurene.

Here, we comment on the uniqueness of Te in exhibiting such a transition from large polaron to small polaron with thickness reduction. Small polarons require a polar phonon and strong electron-phonon interaction. They have been observed in a number of materials such as transition metal oxides (*39*), organic semiconductors (*40*), 2D materials (*41*, *42*), etc. Transitions between large and small polarons have also been observed in perovskite manganese oxides (*12*, *13*) like La_0.75_Ca_0.25_MnO_3_ through the metal-semiconductor/insulator transition with the change of doping or temperature. Bulk Te has a nonpolar A_1_ phonon, while this A_1_ phonon becomes polar with reduced thickness. Such a polar phonon is important in forming strong electron-phonon interaction that leads to small polarons. The transition from nonpolar to polar phonon with reduced thickness is due to the change of nearest-neighbor chains of Te. Because Te is a quasi-1D structure as confirmed by magneto-transport and strain-dependent Raman measurements (*20*), the interactions for one chain with adjacent in-plane or out-of-plane chains are equal in bulk tellurium. This is different from conventional 2D materials where the phonon polarity does not change as thickness varies because the in-plane bond is much stronger than the out-of-plane van der Waals bond. Overall, the unique quasi-1D structure of Te and the A_1_ vibrational profile leads to a change of A_1_ polarity with different thicknesses, eventually resulting in the transition from large to small polarons and modulation of phonon frequency and linewidth.

### Structural changes in interchain and intrachain distances

**Fig. 4.**
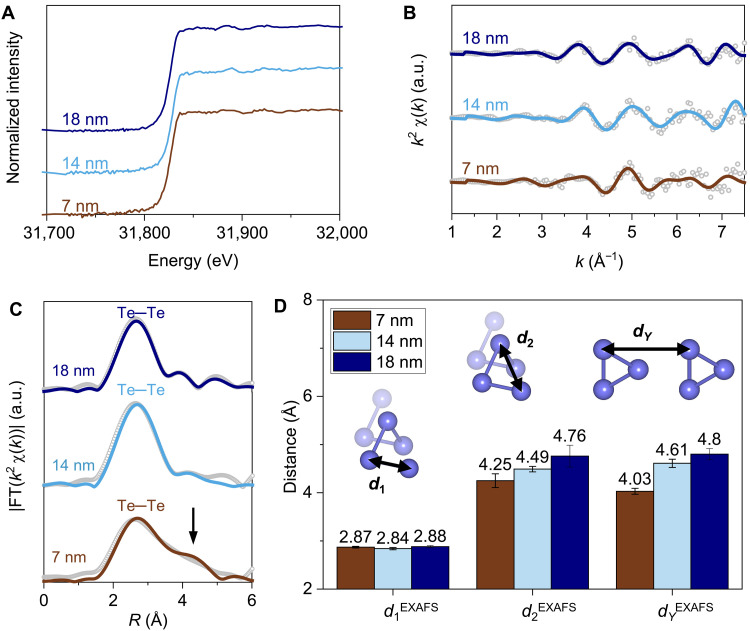
X-ray absorption spectra of tellurene for three different thicknesses. (**A**) Normalized x-ray near-edge fine structure spectra of tellurene for three different thicknesses at Te K-edge. The x-ray absorption spectra are shifted in the *y* axis to show the full spectra. There is negligible energy shift (<3 eV) of the absorption edge. (**B**) The EXAFS (open dots) and its fitting (solid curves) at a $k^{2}$-weight and with background subtracted. (**C**) Fourier transform of EXAFS data. (**D**) The intrachain distance between the nearest Te atoms *d*_1_^EXAFS^, the distance between the second nearest Te atoms in a single chain *d*_2_^EXAFS^, and the lateral distance between neighboring chains *d_Y_*^EXAFS^ were obtained from the EXAFS fitting.

Considering the structural evolution associated with the change of tellurene thickness, an effective method to investigate the atomic arrangements in quasi-1D tellurene with varying thicknesses is through EXAFS analysis. The EXAFS analyzes the oscillatory feature in the x-ray absorption spectrum beyond the absorption edge, which reveals the bond distance between the central atom and their neighboring atoms. Figure 4A shows the normalized x-ray absorption spectra at Te K-edge for tellurene with thicknesses of 18, 14, and 7 nm. The absence of the pre-edge feature is consistent with the x-ray absorption near-edge fine structure of bulk tellurium, indicating that the valence of tellurene remains the same for the analyzed thicknesses (*43*). We then extracted the EXAFS spectra (Fig. 4B) and fitted the bond distance based on the structure model in fig. S9. After Fourier transformation, the EXAFS in Fig. 4C shows a major peak located at around 2.8 Å that is assigned to the Te─Te bond between the nearest atoms. This confirms that there is no phase transition present for tellurene films with different thicknesses. However, the subtle difference in the EXAFS data suggests a slight structural distortion for thinner tellurene films below the critical thickness. Compared to the 14- or 18-nm tellurene, tellurene of 7 nm displays an increased amplitude at around 4 to 5 Å in the R-space (Fig. 4C). This suggests an increased scattering of photoexcited electrons by Te atoms in the neighboring chains, indicating the presence of structural changes in tellurene of 7 nm.

The quantitative EXAFS fitting reveals how intrachain and interchain distances vary for different thicknesses (Fig. 4D). For the three thicknesses measured, the average distance between the nearest Te atoms in the same chain, *d*_1_^EXAFS^, is 2.86 Å with a variation of less than 2%. By contrast, the distance between the second nearest Te atoms in the same chain, *d*_2_^EXAFS^, decreases from 4.76 to 4.25 Å for tellurene from 18 to 7 nm, demonstrating the contraction of the helical chain due to the lattice deformation. Most notably, the distance between adjacent chains in the lateral direction *d_Y_*^EXAFS^ reduces by 0.19 Å (4%) from 18 to 14 nm and further by 0.57 Å (12%) from 14 to 7 nm. Contrary to the lateral interchain distance, the distance between chiral chains in the vertical direction shows a slight increase for thinner tellurene of 7 nm as compared to thicker flakes (fig. S9), which is in agreement with our calculations discussed next. The analyzed interchain distances corroborate the structural distortion between neighboring chains promoting the formation of small polarons. As the thickness decreases, tellurium chains in the same plane with respect to the underlying substrate move closer, while tellurium chains in the vertical direction displace further away.

The difference in interchain distance between two thicknesses is consistent with the low-frequency phonon modes representing relative vibrations between Te chains. Similar to high-frequency modes, the low-frequency interchain modes also display a strong dependence on tellurene thickness (fig. S10). Below the critical thickness of 10 nm, the interchain modes gain amplitude and blue shift as shown in fig. S10, suggesting an enhanced interchain coupling strength (*44*, *45*). This can be potentially explained by the relative displacement between the helical chains in the vertical and horizontal directions observed in the EXAFS fitting. Moreover, the anisotropy of the interchain modes is stronger for thinner tellurene (fig. S11), which is also indicative of an interchain restructuring that leads to a stronger phonon anisotropy.

**Fig. 5.**
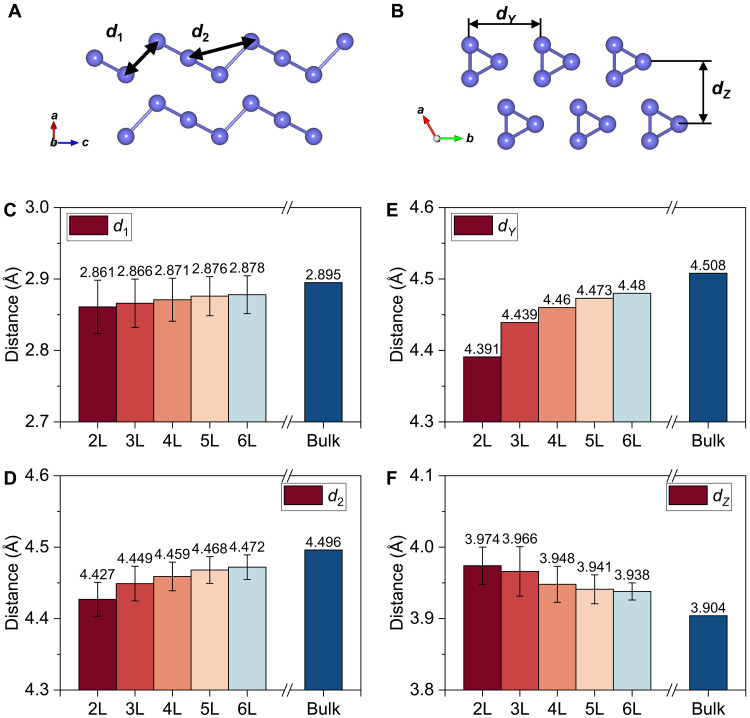
Intrachain and interchain distances by first-principles calculations. (**A**) Schematics of the distance between the nearest Te atoms *d*_1_ and the distance between the second nearest Te atoms in a single chain *d*_2_. (**B**) Horizontal interchain distance *d_Y_* and vertical interchain distance *d_Z_*. (**C** to **F**) The intrachain distances (C) *d*_1_ and (D) *d*_2_, and interchain distances (E) *d_Y_* and (F) *d_Z_* are computed by first-principles calculations for tellurene with different layer numbers. The error bar represents the SD within one unit cell.

We then compare the experimental bond distance to the computed crystal structure for few-layer tellurene based on DFT calculations. The calculated atomic distances within the same chain and between adjacent chains were averaged within the unit cell (Fig. 5, A and B, and fig. S12). The intrachain distance *d*_1_ and *d*_2_ are reduced by approximately 1% from six layers to two layers (Fig. 5, C and D), consistent with the bond distances obtained from EXAFS fitting and calculated bond distances in literature (*46*). In comparison, the lateral interchain distance shortens for thinner tellurene as shown by the computed lattice constant in Fig. 5E. This is again in agreement with the smaller *d_Y_*^EXAFS^ for the 7-nm tellurene than that of the 18-nm tellurene in the EXAFS measurement. The increase of the interchain separation in the vertical direction obtained from EXAFS is reproduced by calculation (Fig. 5D). The lateral contraction and the vertical expansion revealed by the calculations support our EXAFS analysis where few-layer tellurene behaves more like a layered material rather than bulk materials composed of 1D atomic chains. This is in agreement with prior reports where few-layer tellurene can exhibit a covalent-like quasi-bonding between neighboring chains (*36*). Such structural uniqueness contributes to the formation of polar phonons and hence small polarons in few-layer tellurene.

## DISCUSSION

In summary, we investigated the unique behavior of the A_1_ phonon in quasi-1D tellurene structures of varying thicknesses. A pronounced blue shift and broadening were observed for the A_1_ phonon as tellurene thins down, indicating a transition from large polarons in bulk tellurium to small polarons in few-layer tellurene. The increasing polar nature of the A_1_ phonon, predicted by first-principles calculations, further substantiates the formation of small polarons with reduced thickness. This transition from nonpolar to polar phonons in quasi-1D tellurene is fundamentally related to changes in the nearest-neighbor chains of tellurene. Unlike conventional 2D materials, where phonon polarity remains stable with varied thicknesses because of strong in-plane bonds, the quasi-1D structure allows for modulation in phonon characteristics across thicknesses. This structural evolution influences electron-phonon interactions, leading to the formation of small polarons characterized by localized charge carriers and reduced mobility. Our theoretical model elucidates the phonon hardening and broadening effects due to polaron formation, providing insights into the underlying electron-phonon interactions. We then experimentally and theoretically validated the structural modification in tellurene with a varied number of layers. The EXAFS spectroscopy revealed a reduction in the lateral distance between neighboring helical chains of tellurene atoms for smaller thicknesses, aligning with the crossover between large polarons and small polarons. Our research contributes to a deeper understanding of the relationship between polarons and phonon properties in low-dimensional elemental materials. Future investigations could explore the implications of small-polaron formation on device performance and the potential of tellurene-based materials for advanced functional devices.

## MATERIALS AND METHODS

### Raman spectroscopy

The Raman spectra were measured on a Horiba LabRAM spectrometer at room temperature, which was equipped with an ultralow-frequency filter module for low-frequency measurement. The 532-nm laser beam was incident and collected through a 100× objective with a spot size of 1 μm^2^. To avoid beam damage, the laser power was kept below 40 μW. The polarization-resolved measurement was performed with a polarizer added to the detection path of the spectrometer. The differential reflectance spectra were measured with the Laser-Driven Light Sources as the broadband white light.

### X-ray absorption spectroscopy

EXAFS analysis was performed on tellurene to investigate their local atomic structure. The tellurene flakes of varied thickness were deposited on Kapton film. EXAFS measurements were conducted at the Te K-edge (31.814 keV) using synchrotron radiation at 20-BM at the Advanced Photon Source, Argonne National Laboratory. The incident x-ray beam of 500 × 500 μm^2^ was collimated to ~20 × 20 μm^2^, and the samples were scanned to find individual tellurene flakes. The monochromator was detuned by 15% to reduce x-rays with higher harmonic energies. EXAFS spectra were collected in transmission mode using ionization chambers filled with argon gas. The spectra were analyzed using the Athena and Artemis software packages (*47*). The absorption spectra were processed by removing the pre-edge and normalized to the edge step, followed by standard background removal and Fourier transformed to R-space for EXAFS modeling. The ultrathin Te samples make it very challenging to obtain high data quality at high *k* space. The detailed fitting results can be found in table S1.

### Analytical theory

The renormalized phonon frequency could be calculated through the equation of motion method for the phonon’s Green’s function. If we perform the perturbation of the hopping amplitude under the proper assumption, we can simplify the Hamiltonian and calculate the phonon linewidth on the basis of the one-loop polaron correction. The detailed derivation of the comprehensive analytical theory can be found in the Supplementary Materials.

### First-principles calculations

First-principles calculations were carried out using the Vienna ab initio simulation package (VASP v.5.4.4) with projector augmented-wave pseudopotentials for electron-ion interactions (*48*) and the Perdew-Burke-Ernzerhof functional for exchange-correlation interactions (*49*). For bulk Te, both atoms and cell volume were allowed to relax until the residual forces were below 0.001 eV/Å, with a cutoff energy set at 300 eV and a gamma-centered 18 × 18 × 14 k-point sampling. The optimized lattice constants were *a* = *b* = 4.508 Å, and *c* = 5.959 Å, where the chiral Te chain direction is along the *c* direction (see Fig. 1A). Few-layer Te systems were then modeled by a periodic slab geometry, where a vacuum separation of 22 Å in the out-of-plane direction was used to avoid spurious interactions with periodic images. As a common practice, the out-of-plane direction is defined as the *z* direction. For the 2D slab calculations, the chiral Te chain direction is defined as the *x* direction while the interchain direction is along the *y* axis, where 14 × 18 × 1 k-point samplings were used. All atoms were relaxed until the residual forces were below 0.001 eV/Å and the in-plane lattice constants were also optimized. Phonon calculations were performed using the optimized structures. The dynamic matrix was calculated using the finite difference scheme implemented in the Phonopy software (*50*). Hellmann-Feynman forces in the supercell (3 × 3 × 2 for the bulk and 2 × 3 × 1 for few-layer systems) were computed by VASP for both positive and negative atomic displacements (δ = 0.03 Å) and then used in Phonopy to construct the dynamic matrix, whose diagonalization provides phonon frequencies and phonon eigenvectors (i.e., vibrations). After obtaining the eigenvectors of phonon modes in the bulk and few-layer Te systems, we then calculated the change of the dipole moment introduced by a phonon vibration. For the system in equilibrium, we computed the electric dipole moment using the Berry phase method (*51*) and then introduced perturbation into the atomic structure by the amount of the phonon eigendisplacements (i.e., phonon eigenvectors normalized by the atomic mass) and recomputed the dipole moment. The difference of the dipole moment between the equilibrium and perturbed structures is thus the change of the dipole moment by the phonon vibration (*52*–*54*).
