# Supplementary material for: Thickness-dependent polaron crossover in tellurene
Source: Sci Adv. 2025 Jan 8;11(2):eads4763. doi: 10.1126/sciadv.ads4763 (PMC11708887; doi:10.1126/sciadv.ads4763)
Supplement: Supplementary file 1 — Supplementary Text Figs. S1 to S13 Table S1 [file sciadv.ads4763_sm.pdf]

Science Advances
AAAS

Supplementary Materials for

Thickness-dependent polaron crossover in tellurene

Kunyan Zhang *et al.*

Corresponding author: Shengxi Huang, shengxi.huang@rice.edu; Mingda Li, mingda@mit.edu

Sci. Adv. **11**, eads4763 (2025)
DOI: 10.1126/sciadv.ads4763

This PDF file includes:

Supplementary Text
Figs. S1 to S13
Table S1

Supplementary Text

<u>Supplementary Note 1.</u> Breit-Wigner-Fano (BWF) line shape.

The Breit-Wigner-Fano (BWF) line shape is defined in this paper as

$$I(\omega) = I_0 \frac{[1 + (\omega - \omega_0)/(q_s\Gamma_0/2)]^2}{1 + [(\omega - \omega_0)/(\Gamma_0/2)]^2} \quad (1)$$

in which I_0, ω_0, and Γ_0 denote the intensity, frequency, and linewidth of the modified discrete state, respectively. The asymmetry parameter $1/q_s$ quantifies the level of Fano resonance.

We chose the BWF function to fit the Raman spectra because it captures the asymmetric lineshape that arises from Fano resonance. **Fig. S3** presents a comparison between fittings of the A_1 mode using BWF, Lorentzian, and Gaussian functions. For all thicknesses considered, the fitting using a BWF function for the A_1 phonon renders an R^2 greater than 0.99, making the BWF function the most accurate model for fitting and interpreting these features. In contrast, fitting using a Lorentzian or Gaussian function underestimates the right side of the A_1 mode for most thicknesses, yielding a lower R^2.

<u>Supplementary Note 2.</u> Polaron theory.

We begin with the general electron-phonon interacting Hamiltonian, assuming spinless electrons and also single-mode phonons with normalized $\hbar = 1$,

$$H = H_{0\mathrm{e}} + H_{0\mathrm{ph}} + H_{\mathrm{e-ph}} = -t\sum_{\langle ij\rangle} c_i^{\dagger} c_j + \sum_{\mathbf{q}} \omega_{\mathbf{q}}\, a_{\mathbf{q}}^{\dagger} a_{\mathbf{q}} + \sum_{j\mathbf{q}} g_{\mathbf{q}} c_j^{\dagger} c_j \left(a_{\mathbf{q}} + a_{-\mathbf{q}}^{\dagger}\right) e^{i\mathbf{q}\cdot\mathbf{R}_j}, \quad (1)$$

$g_{\mathbf{q}}$ is the electron-phonon coupling constant where the detailed form depends on the type of electron-phonon interactions (*37*), the notation $\langle ij\rangle$ indicates that sites i and j are nearest neighbors. $H_0 = H_{0\mathrm{e}} + H_{0\mathrm{ph}}$ is the non-interacting Hamiltonian for electron and phonon, respectively. H_{e-ph} is the electron-phonon interaction term. This model describes the coupling between local polarization at site *j* with the electron density.

Large polaron model:

For weak coupling between electrons and phonons, with large electron bandwidth, we can solve it in momentum space. Defining $c_{\mathbf{k}} = \frac{1}{\sqrt{N}}\sum_j c_j e^{-i\mathbf{k}\cdot\mathbf{R}_{\mathrm{j}}}$, we have

$$H_0 = -t\sum_{\mathbf{k}} W_{\mathbf{k}} c_{\mathbf{k}}^{+} c_{\mathbf{k}} + \sum_{\mathbf{q}} \omega_{\mathbf{q}} \left(a_{\mathbf{q}}^{+} a_{\mathbf{q}} + \frac{1}{2}\right), \quad (2)$$

$$H_{\text{e-ph}} = \sum_{\mathbf{kq}} g_{\mathbf{q}} c_{\mathbf{k+q}}^{+} c_{\mathbf{k}} \left(a_{\mathbf{q}} + a_{-\mathbf{q}}^{+}\right), \quad (3)$$

where $W_{\mathbf{k}} = \sum_{NN} e^{i\mathbf{k}\cdot\mathbf{R}_{\mathrm{NN}}}$, and defining $\varepsilon_{\mathbf{k}} = tW_{\mathbf{k}}$ and $\mathbf{R}_{\mathrm{NN}} = \mathbf{R}_{\mathrm{i}} - \mathbf{R}_{\mathrm{j}}$is the relative distance for nearest neighbors. Defining the phonon propagator as

$$D(\mathbf{q},\tau) = -T_\tau \left\langle \left(a_{\mathbf{q}}^{+}(\tau) + a_{-\mathbf{q}}(\tau)\right)\left(a_{-\mathbf{q}}^{+}(0) + a_{\mathbf{q}}(0)\right)\right\rangle; D(\mathbf{q}, i\omega_n) = \int_0^{\beta} D(\mathbf{q},\tau) e^{+i\omega_n\tau} d\tau. \quad (4)$$

For non-interacting phonons, we have $D^{(0)}(\mathbf{q}, i\omega_n) = \frac{2\omega_q}{(i\omega_n)^2 - \omega_{\mathbf{q}}^2}$. With electron-phonon interaction, the polarization operator can be written as

$$\Pi^{(1)}(\mathbf{q}, i\omega_n) = \frac{1}{\beta V}\sum_{\mathbf{p}, ip_n} G^{(0)}(\mathbf{p}, ip_n) G^{(0)}(\mathbf{p}+\mathbf{q}, ip_n + i\omega_n). \quad (5)$$

The renormalized phonon Green's function can be written as

$$D(\mathbf{q}, i\omega_n) = \frac{2\omega_{\mathbf{q}}}{(i\omega_n)^2 - \omega_{\mathbf{q}}^2 - 2\omega_{\mathbf{q}}(g_{\mathbf{q}})^2 \Pi^{(1)}(\mathbf{q}, i\omega_n)}. \quad (6)$$

The renormalized phonon frequency and lifetime can be solved as the poles,

$$\left(\Omega_{\mathbf{q}} - i\Gamma_{\mathbf{q}}\right)^2 = \omega_{\mathbf{q}}^2 + 2\omega_{\mathbf{q}} \left|g_{\mathbf{q}}\right|^2 \Pi^{(1)}(\mathbf{q}, i\omega_n), \quad (7)$$

which, given the small electron-phonon coupling constant $g_{\mathbf{q}}$**,** the modulation to phonon frequency in the weak coupling regime is negligible. While the phonon linewidth under condition $\mathbf{q} \to 0$, we have $\Gamma_0 = -\frac{\omega_0}{\Omega_0}\left|g_{\mathbf{q}}\right|^2 \mathrm{Im}\Pi^{(1)}(\mathbf{q} \to 0, \omega_0) \to 0$, ω_0 is the unnormalized frequency when $\mathbf{q} \to 0$.

Small polaron model for phonon frequency renormalization:

After performing the small polaron where the polaron is more localized in real space and electrons are strongly coupled to the phonons, the so-called Lang-Firsov unitary transformation (*37*), finally the Hamiltonian after canonical transform can be rewritten as

$$H = -\sum_{j} E_B c_j^{+} c_j - t \sum_{\langle ij \rangle} exp\left(-\sum_{\mathbf{q}} T_{\mathbf{q}ij} p_{\mathbf{q}} e^{-i\mathbf{q}\cdot\mathbf{R}_i} \right) c_i^{+} c_j + \sum_{\mathbf{q}} \omega_{\mathbf{q}} a_{\mathbf{q}}^{+} a_{\mathbf{q}}, \qquad (8)$$

where the canonical phonon operators are defined in convention as $u_{\mathbf{q}} = \sqrt{\frac{\hbar}{2m\omega_{\mathbf{q}}}} (a_{\mathbf{q}} + a_{-\mathbf{q}}^{\pm})$ and $p_{\mathbf{q}} = i\sqrt{\frac{\hbar m \omega_{\mathbf{q}}}{2}} (a_{\mathbf{q}}^{+} - a_{-\mathbf{q}})$, $T_{\mathbf{q}ij} = \frac{g_{\mathbf{q}}^{*}}{\omega_{\mathbf{q}}} i \sqrt{\frac{2}{\hbar m \omega_{\mathbf{q}}}} (1 - e^{i\mathbf{q}\cdot(\mathbf{R}_i - \mathbf{R}_j)})$, $E_B = \sum_{\mathbf{q}} \frac{|g_{\mathbf{q}}|^2}{\omega_{\mathbf{q}}}$ is the normalized small polaron binding energy. The small polaron can lead to phonon hardening, which is shown in Ref. (*38*) for instance. Through the standard procedure of the equation of motion for the phonon Green's function, the renormalized phonon frequency can be written as

$$\Omega_{\mathbf{q}} = \omega_{\mathbf{q}} \left(1 + \frac{4|g_{\mathbf{q}}|^2}{\omega_{\mathbf{q}}^2 N} \sum_{\mathbf{k}n} \frac{te^{-S_n}}{\omega_{\mathbf{q}}} (1 - cos\, \mathbf{q} \cdot \mathbf{R}_n) n_{F\mathbf{k}} e^{+i\mathbf{k}\cdot\mathbf{R}_n} \right), \qquad (9)$$

where $S_n = \frac{2}{N} \sum_{\mathbf{q}} \frac{|g_{\mathbf{q}}|^2}{\omega_{\mathbf{q}}^2} sin^2\left(\frac{\mathbf{q}\cdot\mathbf{R}_n}{2}\right)(2n_{B\mathbf{q}} + 1)$. e^{-S_n} is the effective factor of the electron hopping, which reflects the effective localization of the small polaron and leads to the reduction of the electrical mobility. Based on Holstein's original estimation (*3*), the range of the value for e^{-S_n} is $10^{-2} \sim 10^{-4}$, we take $e^{-S_n} = 10^{-3}$ during the numerical calculation. Notice that the effective hopping factor e^{-S_n} also have the temperature dependence. However, the non-interacting phonon frequency before the normalization also has an approximately linear temperature dependence indicated by the experimental data, this makes the factor in the Boson statistics, $\frac{\hbar\omega}{kT}$, becomes weakly dependent on the temperature. As a result, we assume the value of the effective hopping factor remains relatively stable around room temperature. Besides, applying the linear temperature dependence for factor S will cause a large change in the frequency renormalization due to the exponential behavior, which violates the experimental observation of the temperature dependence of the phonon frequency.

For the polar phonon, we take the form of $g_{\mathbf{q}} = \frac{\sqrt{2\pi e^2 \omega_{\mathbf{q}} \left(\frac{1}{\varepsilon_\infty} - \frac{1}{\varepsilon_0}\right)}}{\mathbf{q}}$ (*1*), and e is the elementary charge. From the previous literature (*52*), we can get the layer-dependent dielectric constant, and we take the empirical approximation that $\varepsilon_\infty = 0.5\varepsilon_0$, here ε_0 and ε_∞ are dielectric constant for static case and infinity frequency, respectively. The static dielectric constant of two to six layers along the $E \perp$ C direction from the literature mentioned above is taken as 10.3, 14.3, 20, 21.4, and 27.3, respectively [4]. The phonon frequency simulated using the static dielectric constant along the $E \parallel$ C direction is consistent with the results based on the static dielectric constant along the $E \perp$ C direction. We set the chemical potential as 0.75 eV based on the literature (*53, 54*), assuming the electron hopping as $t = 0.21\hbar\omega_{\mathbf{q}}(\mathbf{q} \to 0)$ and assuming $\omega_{\mathbf{q}}(\mathbf{q} \to 0) = 121\ \mathrm{cm}^{-1}$ for the A_1

phonon, with the first nearest neighbor distance being 0.289 nm based on our EXAFS measurement presented in **Fig. S9**, we finally can get the numerically calculated results presented in the main content. For the temperature-dependent frequency calculations in **Fig. S8**(b), the static dielectric constant is taken as 14.3, and the phonon frequency before the normalization $\omega_{\mathbf{q}}$ is assumed to be linearly dependent on the temperature from 125.84 cm^{-1} to 121 cm^{-1} within the temperature range from 80 K to 300 K. If we set $F_n = \frac{4|g_{\mathbf{q}}|^2}{\omega_{\mathbf{q}}^2 N}\sum_{\mathbf{k}}\frac{te^{-S_n}}{\omega_{\mathbf{q}}}(1 - cos\, \mathbf{q}\cdot\mathbf{R_n})n_{F\mathbf{k}}e^{+i\mathbf{k}\cdot\mathbf{R_n}}$ as the phonon hardening factor, we can also observe a dependence of phonon frequency with various factors such as the static dielectric constant, the nearest neighbor distance, and the chemical potential (see **Fig. S13**). The static dielectric constant for (b) and (c) is assumed as 8, and other parameters are taken as mentioned before.

From **Fig. S13(b)**, we noticed that the enhancement factor is rapidly suppressed as the Te-Te distance increases. This indicates the first-nearest neighbors dominate the contribution of the phonon frequency renormalization. The hopping amplitudes for second-nearest neighbors and other more distant sites are generally small and can be neglected. As a result, we only need to consider the first-nearest neighbor hopping into the Hamiltonian whose contribution dominates. On the other hand, the change of the intrachain second-nearest atomic distances and the lateral interchain distances causes the change of the dipole moment and then the dielectric constant. Therefore, by modifying the dielectric properties (appeared in $g_{\mathbf{q}}$ of Eq. 3 and explicitly in Eq. 4), the change of these atomic distances indirectly affects the polaron crossover.

Small polaron model for phonon linewidth:

We perform the perturbation of the hopping amplitude, $t_{ij} = t\, exp\left(-\sum_{\mathbf{q}} T_{\mathbf{q}ij}p_{\mathbf{q}}e^{-i\mathbf{q}\cdot\mathbf{R}_\mathrm{i}}\right) \approx t\left(1 - \sum_{\mathbf{q}} T_{\mathbf{q}ij}p_{\mathbf{q}}e^{-i\mathbf{q}\cdot\mathbf{R}_\mathrm{i}}\right)$, which is valid when $-\sum_{\mathbf{q}} T_{\mathbf{q}ij}p_{\mathbf{q}}e^{-i\mathbf{q}\cdot\mathbf{R}_\mathrm{i}}$ is sufficiently small. This should be satisfied as in large **q**, $g_{\mathbf{q}}^*$ should have the $1/q$ dependence and will lead to sufficient small $g_{\mathbf{q}}^*$. For small **q**, the $1/q$ dependence will cancel with the **q** dependence from $\left(1 - e^{i\mathbf{q}\cdot\mathbf{R}_\mathrm{i}}\right)$ within the $T_{\mathbf{q}ij}$. Besides, as we are taking the nearest neighbor, we can assume $\mathbf{R}_\mathrm{i} - \mathbf{R}_\mathrm{j}$ is small enough to lead to valid perturbation expansion.

Instead of the lattice displacement $u_{\mathbf{q}}$ and strength $g_{\mathbf{q}}$ in conventional electron-phonon coupling, the electrons in this regime couple with lattice momentum $p_{\mathbf{q}}$ with strength $\frac{g_{\mathbf{q}}^*}{\omega_{\mathbf{q}}}$. It is feasible to calculate the polaron "polarization" operator that renormalizes phonon energy and gains phonon the lifetime, through the phonon momentum-momentum correlator. Performing the Fourier transform $c_i = \frac{1}{\sqrt{N}}\sum_{\mathbf{k}} e^{-i\mathbf{k}\cdot\mathbf{R}_\mathrm{i}}c_{\mathbf{k}}$, the Hamiltonian becomes

$$H' = -\sum_{\mathbf{k}}\left(E_B + t\sum_{n} e^{i\mathbf{k}\cdot\mathbf{R_n}}\right)c_{\mathbf{k}}^{+}\, c_{\mathbf{k}} - t\sum_{\mathbf{kq}} g_{\mathbf{q},\mathbf{k},\mathrm{eff}}c_{\mathbf{k+q}}^{+}c_{\mathbf{k}}\left(a_{\mathbf{q}}^{+} - a_{-\mathbf{q}}\right) + \sum_{\mathbf{q}} \omega_{\mathbf{q}}a_{\mathbf{q}}^{+}a_{\mathbf{q}}, \quad (10)$$

where “**n**” means the summation of the nearest neighbors, $\mathbf{R_n}$ is the relative distance between two nearest neighbors, and $g_{\mathbf{q},\mathbf{k},\mathrm{eff}} = \sum_{\boldsymbol{n}} \frac{g_{\mathbf{q}}^*}{\omega_{\mathbf{q}}}\left(1 - e^{i\mathbf{q}\cdot\mathbf{R_n}}\right) e^{i\mathbf{k}\cdot\mathbf{R_n}}$.

To proceed, we define the dimensionless momentum self-correlation as

$$F(\mathbf{q},\tau) = -T_\tau \left\langle \left(a_{\mathbf{q}}^{+}(\tau) - a_{-\mathbf{q}}(\tau)\right)\left(a_{-\mathbf{q}}^{\pm}(0) - a_{\mathbf{q}}(0)\right)\right\rangle; F(\mathbf{q}, i\omega_n) = \int_0^\beta F(\mathbf{q},\tau) e^{+i\omega_n \tau} d\tau. \quad (11)$$

Then, using the fact that $a_{\mathbf{q}}(\tau) = a_{\mathbf{q}} e^{-\tau\omega_{\mathbf{q}}}$, we have $F^{(0)}(\mathbf{q}, i\omega_n) = -\frac{2\omega_{\mathbf{q}}}{(i\omega_n)^2 - \omega_{\mathbf{q}}^2} = -D^{(0)}(\mathbf{q}, i\omega_n)$. The free-polaron propagator can be written as $G^{(0)}(\mathbf{p}, ip_m) = \frac{1}{ip_m - (E_B + t\sum_{\boldsymbol{n}} e^{i\mathbf{p}\cdot\mathbf{R_n}})}$, and the 1-loop polaron “polarization operator” can be written as

$$\Pi^{(1)}(\mathbf{q}, i\omega_n) = \frac{t^2}{\beta V}\sum_{\mathbf{p}, ip_n} G^{(0)}(\mathbf{p}, ip_n) G^{(0)}(\mathbf{p}+\mathbf{q}, ip_n + i\omega_n)$$
$$= \frac{t^2}{V}\sum_{\mathbf{p}} \frac{n_F(\varepsilon_{\mathbf{p}}) - n_F(\varepsilon_{\mathbf{p}+\mathbf{q}})}{i\omega_n + \varepsilon_{\boldsymbol{p}} - \varepsilon_{\mathbf{p}+\mathbf{q}}} g_{\mathbf{q},\mathbf{p},\mathrm{eff}}\, g_{\mathbf{q},\mathbf{p}+\mathbf{q},\mathrm{eff}}^*, \quad (12)$$

where $\varepsilon_{\mathbf{p}} = E_B + t\sum_{\boldsymbol{n}} e^{i\mathbf{p}\cdot\mathbf{R_n}}$ is the effective polaron eigenenergy. The renormalized phonon Green’s function can be written based on the momentum-momentum Green’s function as

$$F(\mathbf{q}, i\omega_n) = -\frac{2\omega_{\mathbf{q}}}{(i\omega_n)^2 - \omega_{\mathbf{q}}^2 - 2\omega_{\mathbf{q}}\Pi^{(1)}(\mathbf{q}, i\omega_n)}. \quad (13)$$

The renormalized phonon frequency and lifetime can be solved as the poles. For thicker films, the band structure calculations indicate more semi-metallic behaviors, which has a larger dielectric screening effect. In this regime, we have $g_{\mathbf{q}}^* \propto$ constant, therefore, $g_{\mathbf{q}\to 0,\mathbf{k},\mathrm{eff}} = \sum_{\boldsymbol{n}} \frac{g_{\mathbf{q}}^*}{\omega_{\mathbf{q}}}\left(1 - e^{i\mathbf{q}\cdot\mathbf{R_n}}\right) e^{i\mathbf{k}\cdot\mathbf{R_n}} \to 0$, since the $1 - e^{i\mathbf{q}\cdot\mathbf{R_n}}$ term will be zero at the wavevector $\boldsymbol{q} \to 0$. Therefore, a key feature of the polaron-induced phonon hardening is the lack of the screening effect. The thinner film is semiconducting, contributing to less dielectric screening.

For Raman studies, we can just focus on one specific branch of phonon frequency $\omega_{\mathbf{q}} = \omega_0$, then it appears that $g_{\mathbf{q}\to 0,\mathbf{k},\mathrm{eff}} = \sum_{\boldsymbol{n}} \frac{g_{\mathbf{q}}^*}{\omega_{\mathbf{q}}}\left(1 - e^{i\mathbf{q}\cdot\mathbf{R_n}}\right) e^{i\mathbf{k}\cdot\mathbf{R_n}} \to 0$. However, there is one exception. If there is a polar phonon developed, then the electron-phonon coupling can develop a long-range interaction, i.e., $g_{\mathbf{q}}^* \propto \frac{1}{q}$, resulting in a finite polaron-phonon coupling at the $\mathbf{q} \to 0$ limit. In this case, the effective phonon-polaron coupling constant can be written as (*37*)

$$g_{\mathbf{q}\to 0,\mathbf{k},\mathrm{eff}} = \lim_{\mathbf{q}\to 0}\sum_{\boldsymbol{n}} \sqrt{\frac{2\pi e^2}{\omega_0}\left(\frac{1}{\varepsilon_\infty} - \frac{1}{\varepsilon_0}\right)} \frac{i(\mathbf{q}\cdot\mathbf{R_n})}{q} e^{i\mathbf{k}\cdot\mathbf{R_n}}. \quad (14)$$

Then, for $\mathbf{q} \to 0$ optical phonon, since we are no longer studying metals but more semiconductors, we can generalize from single-band to multi-band, i.e.,

$$\Pi^{(1)}(\mathbf{q}, i\omega_n) = \frac{t^2}{V}\sum_{\mathbf{p}ab}\frac{n_F(\varepsilon_{\mathbf{p}a}) - n_F(\varepsilon_{\mathbf{p+q}b})}{i\omega_n + \varepsilon_{\mathbf{p}a} - \varepsilon_{\mathbf{p+q}b}} g_{\mathbf{q},\mathbf{p},a,\mathrm{eff}} g^*_{\mathbf{q},\mathbf{p+q},b,\mathrm{eff}}, \tag{15}$$

where a and b are band indices. For simplicity, we just use a two-band model. Assuming the initial polaron state is fully occupied $n_F(\varepsilon_{\mathbf{p}a}) = 1$, and the final state is empty $n_F(\varepsilon_{\mathbf{p+q}}) = 0$, and the phonon energy is $\lim_{\mathbf{q}\to 0}\varepsilon_{\mathbf{p+q}b} - \varepsilon_{\mathbf{p}a} \approx \Delta$ as the gap of the polaron band (from occupied to empty band with the assistance of phonons), then we have

$$\Pi^{(1)}(\mathbf{q} \to 0, \omega_0) = \frac{t^2}{V}\sum_{\mathbf{p}}\frac{g_{0,\mathbf{p},\mathrm{eff}} g^*_{0,\mathbf{p},\mathrm{eff}}}{\omega_0 - \Delta + i0^+}, \tag{16}$$

from which we obtain that

$$\mathrm{Im}\Pi^{(1)}(\mathbf{q} \to 0, \omega_0) = -\pi\delta(\omega_0 - \Delta)\frac{2\pi e^2 t^2}{\omega_0}\left(\frac{1}{\varepsilon_\infty} - \frac{1}{\varepsilon_0}\right)\sum_{\boldsymbol{n}}\frac{(\mathbf{q}\cdot\mathbf{R_n})^2}{q^2}. \tag{17}$$

Finally, the phonon linewidth can be written as

$$\Gamma_0 = -\frac{\omega_0}{\Omega_0}\mathrm{Im}\Pi^{(1)}(\mathbf{q} \to 0, \omega_0) = \frac{\omega_0}{\Omega_0}\delta(\omega_0 - \Delta)\frac{2\pi e^2 t^2}{\omega_0}\left(\frac{1}{\varepsilon_\infty} - \frac{1}{\varepsilon_0}\right)\sum_{\boldsymbol{n}}\frac{(\mathbf{q}\cdot\mathbf{R_n})^2}{q^2}. \tag{18}$$

The 1-loop polaron correction diagram contribution will dominate the contribution of the increase of the phonon linewidth, and this gives the results presented in the main text with the parameters mentioned above, and the large polaron linewidth is set to the value of 0.5 cm^{-1}. It is worth mentioning that this polaron gap is not the electronic bandgap. The physical picture under this result indicates that since elemental Te has 3 atoms per unit cell, 2 atoms form the conduction band (CB) and valance band (VB), and the third atom is closer to VB and has a small energy split, assisting the inter-band transition (energy of the A_1 phonon around 15 meV) between the two bands.

Utilizing the polaron theory for both phonon frequency and linewidth, we perform numerical calculations, and the theoretical results presented in the main text exhibit a reasonable agreement with the experimental data. The consistency between our minimal theoretical model and the experimental results showcased the ability of our model to capture the origin of the experimental results and provide a clear physical picture of the polaron crossover. Due to the complexity of possible interactions in the system, we were able to describe these emergent phenomena semi-quantitatively. A more refined model in the future can potentially quantify the hopping mechanism, as well as include other scattering mechanisms and higher-order interactions in detail with more experiments and computational resources.

Small polaron model for transport:
Similarly, the small polaron self-energy can be calculated as (*37*):

$$\Sigma(\mathbf{k}, ik_n) = \frac{t^2}{\beta V} \sum_{\mathbf{q}, i\omega_m} F^{(0)}(\mathbf{q}, i\omega_m) G^{(0)}(\mathbf{k}+\mathbf{q}, ik_n + i\omega_m)$$
$$= \frac{t^2}{V} \sum_{\mathbf{q}} g_{\mathbf{q},\mathbf{k},\mathrm{eff}} g^*_{\mathbf{q},\mathbf{k}+\mathbf{q},\mathrm{eff}} \left[\frac{n_B(\omega_{\mathbf{q}}) + 1 - n_F(\varepsilon_{\mathbf{k}+\mathbf{q}})}{ik_n - \varepsilon_{\mathbf{k}+\mathbf{q}} - \omega_{\mathbf{q}}} + \frac{n_B(\omega_{\mathbf{q}}) + n_F(\varepsilon_{\mathbf{k}+\mathbf{q}})}{ik_n - \varepsilon_{\mathbf{k}+\mathbf{q}} + \omega_{\mathbf{q}}} \right]. \quad (19)$$

The imaginary part of the small polaron self-energy ($\mathbf{q} \to 0$, $\mathrm{g}^*_{\mathbf{q}} \propto \frac{1}{\mathrm{q}}$) related to the phonon scattering is written as:

$$\mathrm{Im}\Sigma(\mathbf{k}, ik_n \to u + i0^+) =$$
$$-\frac{\pi t^2}{V} \sum_{\mathbf{q}} g_{\mathbf{q},\mathbf{k},\mathrm{eff}} g^*_{\mathbf{q},\mathbf{k}+\mathbf{q},\mathrm{eff}} \begin{bmatrix} \left(n_B(\omega_{\mathbf{q}}) + 1 - n_F(\varepsilon_{\mathbf{k}+\mathbf{q}})\right) \delta(\varepsilon_{\mathbf{k}+\mathbf{q}} + \omega_{\mathbf{q}} - u) \\ + \left(n_B(\omega_{\mathbf{q}}) + n_F(\varepsilon_{\mathbf{k}+\mathbf{q}})\right) \delta(\varepsilon_{\mathbf{k}+\mathbf{q}} - \omega_{\mathbf{q}} - u) \end{bmatrix}. \quad (20)$$

The related relaxation time caused by polaron-phonon scattering is:

$$\frac{1}{\tau_{\mathrm{ph}}} = -2\mathrm{Im}\Sigma(\mathbf{k}, u). \quad (21)$$

Notice that the pre-factor of ImΣ under the $\mathbf{q} \to 0$ limit is $2\pi t^2 g_{\mathbf{q}\to 0,\mathbf{k},\mathrm{eff}} g^*_{\mathbf{q}\to 0,\mathbf{k},\mathrm{eff}} \propto \Gamma_0$ relating to the phonon linewidth, and a reduced thickness will increase the effective coupling $g_{\mathbf{q},\mathbf{k},\mathrm{eff}}$ as shown by our experiments and theoretical model. This will increase the electrical resistance due to the polaron-phonon scattering and reduce electron mobility. We acknowledge that thickness effects that do not involve polaron will also affect carrier mobility. For example, the carrier mobility of MoS_2 and black phosphorus first increases and then decreases as a function of increased thickness. This trend is attributed to a reduction in surface scattering and then an enhanced contribution from interlayer resistance, respectively. However, it is important to note that the thickness dependence of carrier mobility in tellurene does not follow this trend, indicating that there are other dominating factors that influence carrier mobility. In our case, we showed that the phonon lifetime and hence carrier mobility is inversely connected to the effective coupling $g_{\mathbf{q},\mathbf{k},\mathrm{eff}}$ which becomes larger at reduced thicknesses.The above discussion considers the small polaron regime where **q** is relatively small, meaning that electron-phonon scattering due to small polarons mostly affects intravalley scattering. For the valley transport between different valleys such as **K** and **K**', the intervalley scattering with polar phonon should be revised as $-t\sum_{\mathbf{k}\mathbf{k}'\mathbf{q}} g_{\mathbf{q},\mathbf{k},\mathrm{eff}} c^+_{\mathbf{k}'} c_{\mathbf{k}} \left(a^+_{\mathbf{q}} - a_{-\mathbf{q}}\right)$, where **k** is around valley **K** and $\mathbf{k}'$ is around valley **K**'. It requires a large phonon wavevector **q** to nest the two valleys and thereby realize the intervalley scattering. While the electron-phonon coupling for polar phonons has the dependence $g^*_{\mathbf{q}} \propto \frac{1}{q}$, it indicates $\mathrm{Im}\Sigma(\mathbf{k}, u) \to 0$ with large phonon wavevector **q**. As a result, small polarons will not significantly affect the valley transport through this polaron-phonon scattering mechanism. This result supports the robustness of valley transport in Te (*19*).

Supplementary Note 3. The theoretical explanation for changes in mobility.

We present the theoretical explanation of the drop in mobility in thinner samples qualitatively. Since the electron mobility μ can be expressed through the electrical conductivity σ:

$$\sigma = \frac{n_0 e^2 \tau}{m_e} = e\mu n_0, \tag{22}$$

where n_0 is the electron density, e is the elementary charge, m_e is the electron mass, τ is the relaxation time. As a result, we should notice that mobility is proportional to the relaxation time. Remind of the Matthiessen's rule, $\frac{1}{\tau} = \frac{1}{\tau_{\text{ele}}} + \frac{1}{\tau_{\text{ph}}} + \frac{1}{\tau_{\text{defect}}} + \cdots,$ and τ_{ele} is the relaxation time caused by electron-electron scattering, τ_{defect} comes from the defect scattering, while τ_{ph} is related to the electron-phonon scattering and should be related to the phonon linewidth: $\frac{1}{\tau_{\text{ph}}} \propto \Gamma_0$. We can see when the thickness of the Te sample is reduced, the increase of the phonon linewidth is confirmed in theory, which leads to the drop in mobility. However, the drop in mobility cannot be attributed solely to the contribution of phonons, even though this theoretical qualitative analysis from the increase of the phonon linewidth matches experimental measurements. How other factors affect mobility is beyond the scope of this theory.

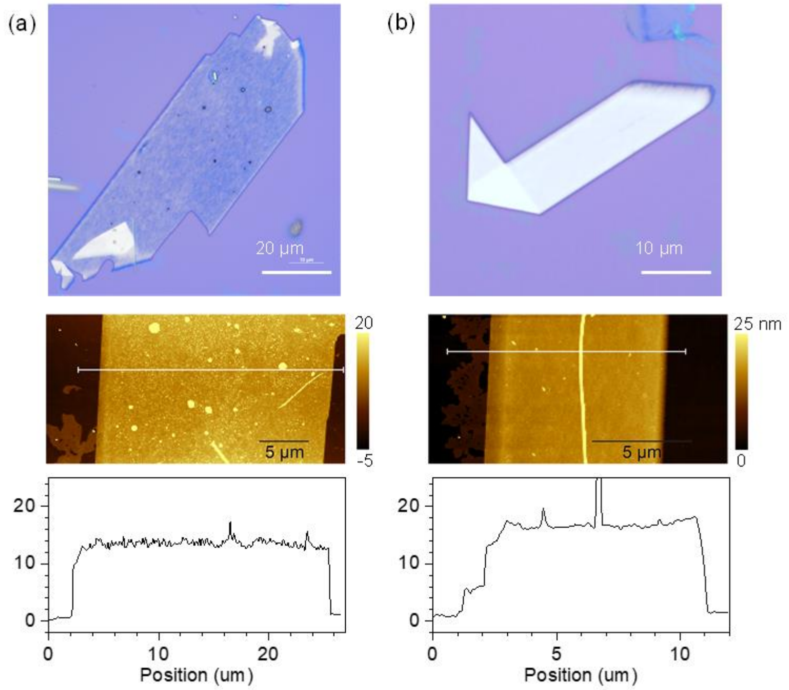

Fig. S1. Atomic force microscopy (AFM) of tellurene with different thicknesses. (a) Thickness of 13.0 nm. (b) Thickness of 15.8 nm. Top panel: optical images. Middle panel: AFM images. Bottom panel: Line scans across the tellurene thin film.

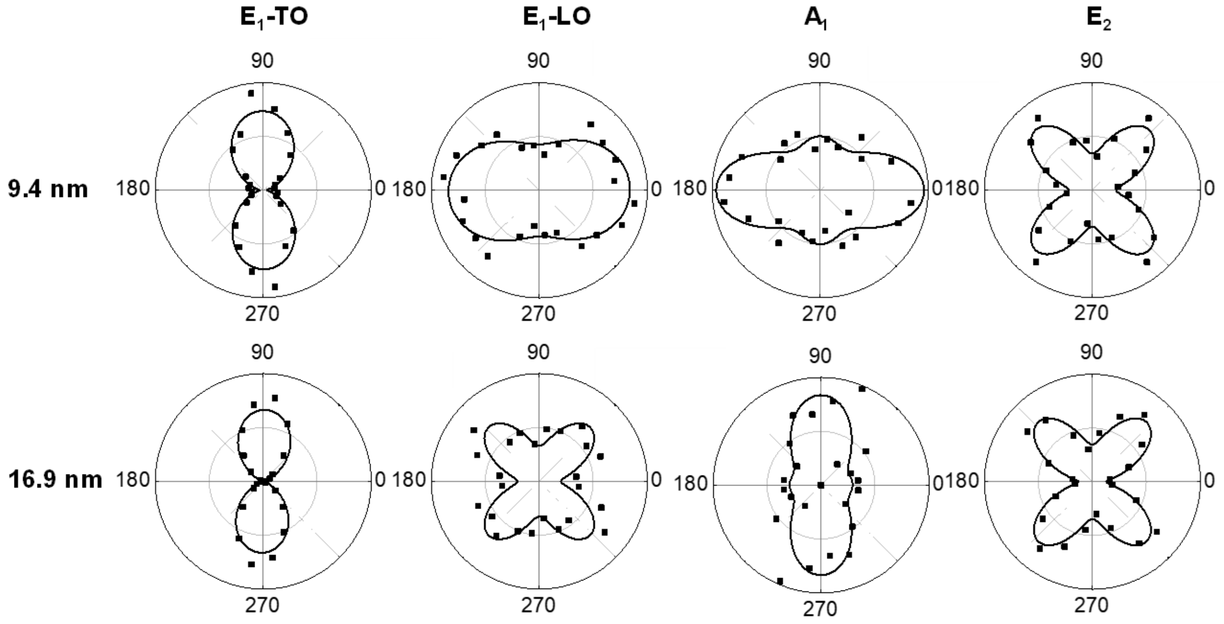

Fig. S2. **Polar plots of Raman intensity**. Raman intensity for tellurene of 9.4 and 16.9 nm including the E_1-TO mode, E_1-LO mode, A_1 mode, and E_2 mode. For the polarization-dependent measurement, the sample was rotated in-plane, and the scattering that is polarized parallel to the incident light was collected. The polarization angle is defined as the angle between the laser polarization and the [0001] direction. Depending on the phonon symmetry of the D_3 point group, the intensity of the characteristic Raman modes changes as a function of the polarization angle. For tellurene with a thickness of 9.1 nm, the A_1 mode exhibits a two-fold symmetry with a maximum intensity at 0° and 180° along the [1000] direction. However, the maximum intensity of the A_1 mode shifts to 90° and 270° when the thickness of tellurene increases to 16.9 nm, indicating changes in the phonon anisotropy. The different anisotropic dependencies can be well explained by the Raman tensor theory. Based on the Raman tensor, the intensity of the A_1 mode can be written as $|\boldsymbol{a}\sin^2\theta + \boldsymbol{b}\cos^2\theta|^2$ in which θ is the polarization angle and $\boldsymbol{a}$ and $\boldsymbol{b}$ are Raman tensor elements. Using this equation, the polar plots of the 9.4 nm tellurene can be fitted with an $\boldsymbol{a}/\boldsymbol{b}$ ratio of 0.86, while the polar plots of the 16.9 nm tellurene can be reproduced with an $\boldsymbol{a}/\boldsymbol{b}$ ratio of 1.18. The change in the $\boldsymbol{a}/\boldsymbol{b}$ ratio directly accounts for the observation that the maximum Raman intensity of the A_1 mode is located at 0° and 180° or 90° and 270° for different thicknesses.

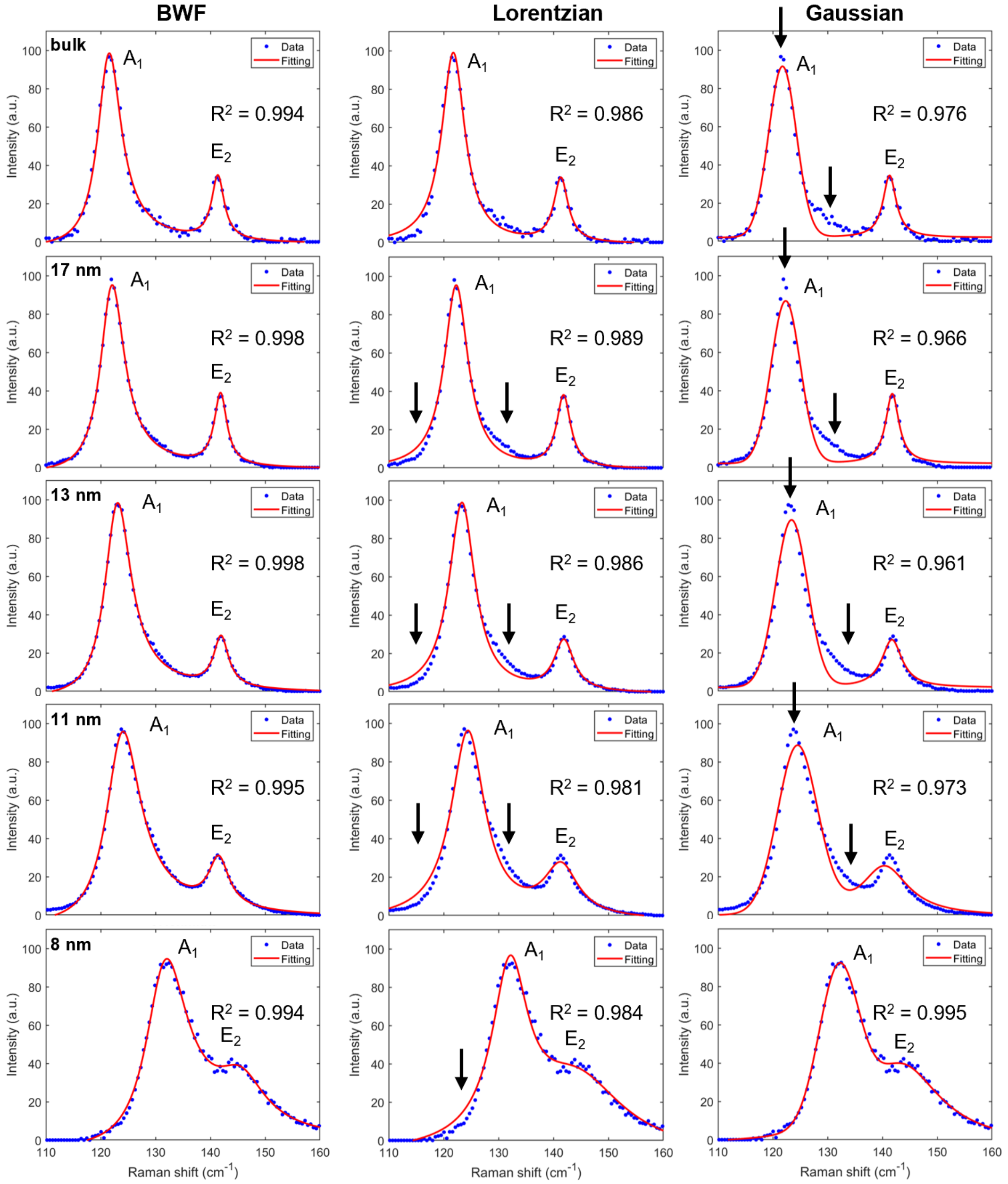

Fig. S3. **Raman spectrum fitting using different functions for the A_1 phonon.** BWF, Lorentzian, and Gaussian functions were used. The Lorentzian and Gaussian functions used for the fitting are expressed as $I(\omega) = I_0 \frac{(\Gamma_0/2)^2}{(\omega-\omega_0)^2+(\Gamma_0/2)^2}$ and $I(\omega) = I_0 \exp\left(-\frac{(\omega-\omega_0)^2}{2{\Gamma_0}^2}\right)$. The E_2 mode was fitted using a Lorentzian function for all three fitting scenarios.

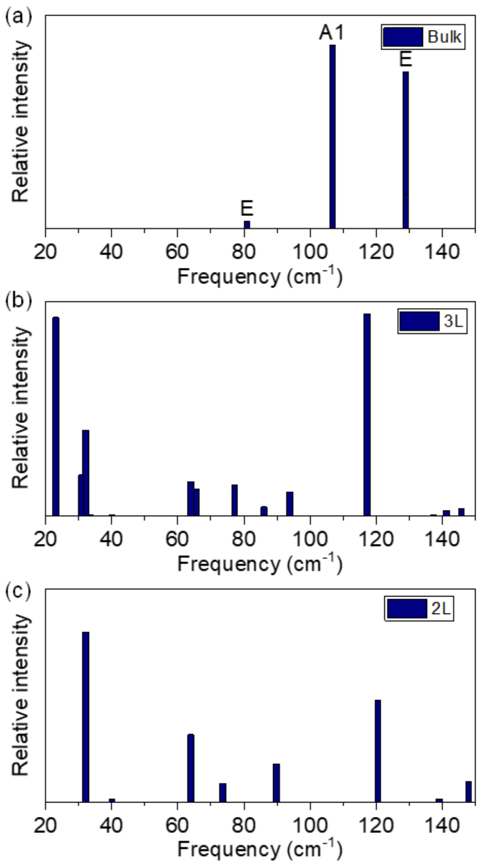

Fig. S4. Calculated Raman intensity. The calculated Raman intensity for (a) bulk tellurium, (b) tellurene composed of three layers of helical chains, and (c) tellurene composed of two layers of helical chains. The tellurene composed of three layers (3L) is predicted to have interchain vibrations at 23.1 cm^{-1} and 30.8 cm^{-1} with relatively strong vibrational amplitudes. While the interchain vibration at 32.1 cm^{-1} is evident for tellurene with two layers (2L).

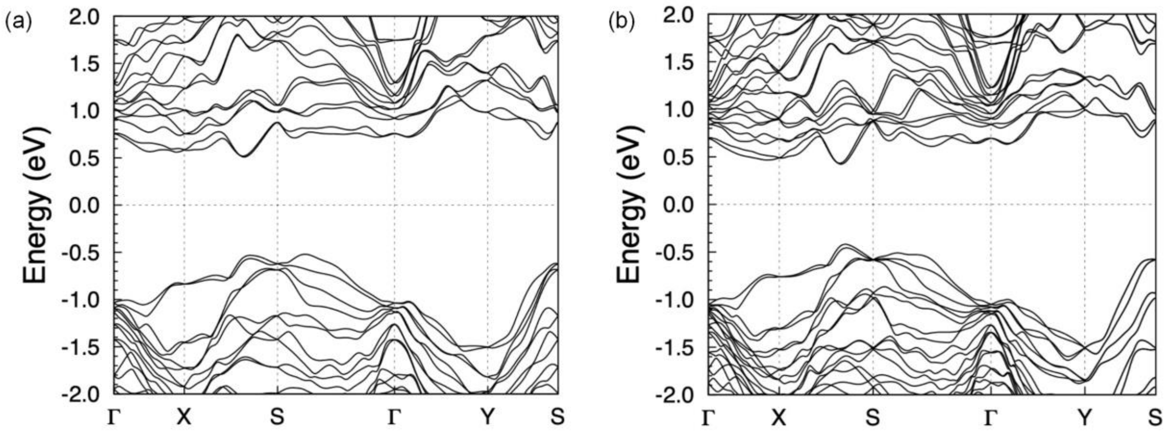

Fig. S5. Calculated band structure. The calculated band structure of (a) 3L and (b) 4L tellurene.

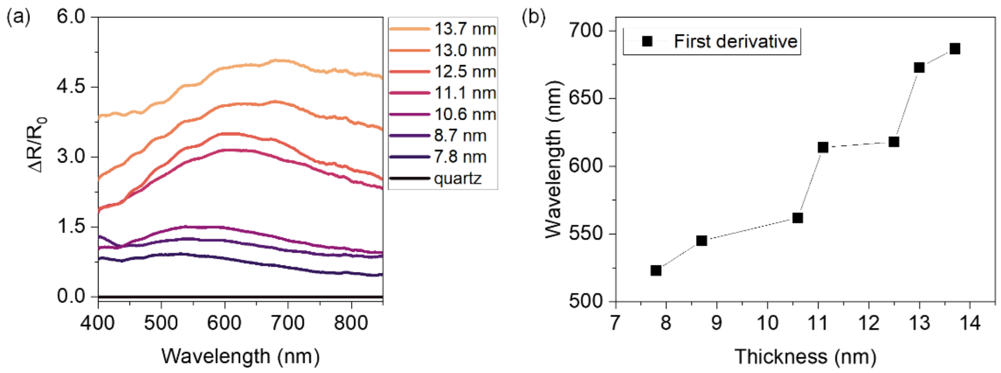

Fig. S6. Differential reflectance of few-layer tellurene on quartz. (a) Differential reflectance spectra of tellurene. $\Delta R/R_0$ is defined as the normalized reflectance difference of tellurene/quartz with respect to quartz. (b) The wavelength where the first derivative of differential reflectance equals zero. This indicates the absorption wavelength of tellurene of different thicknesses.

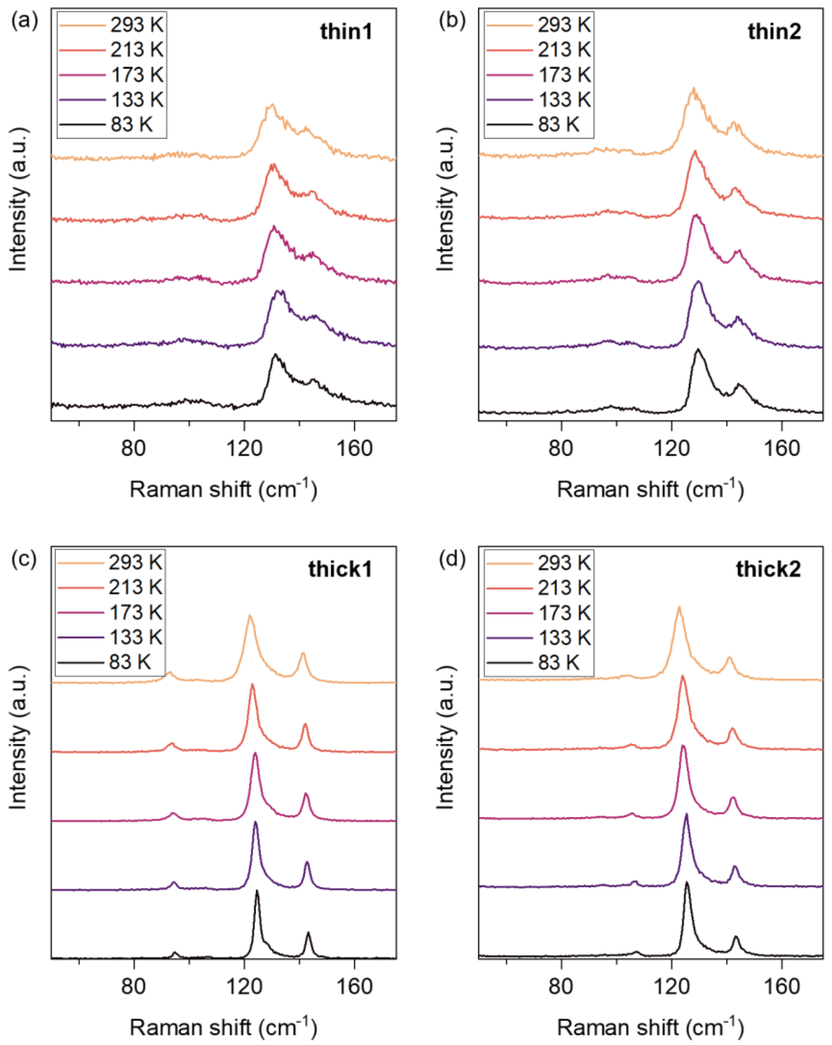

Fig. S7. Temperature-dependent Raman spectra for four Te flakes. The two labeled 'thin' are below the transition thickness, while the two labeled 'thick' are above the transition thickness based on AFM scans and their Raman features.

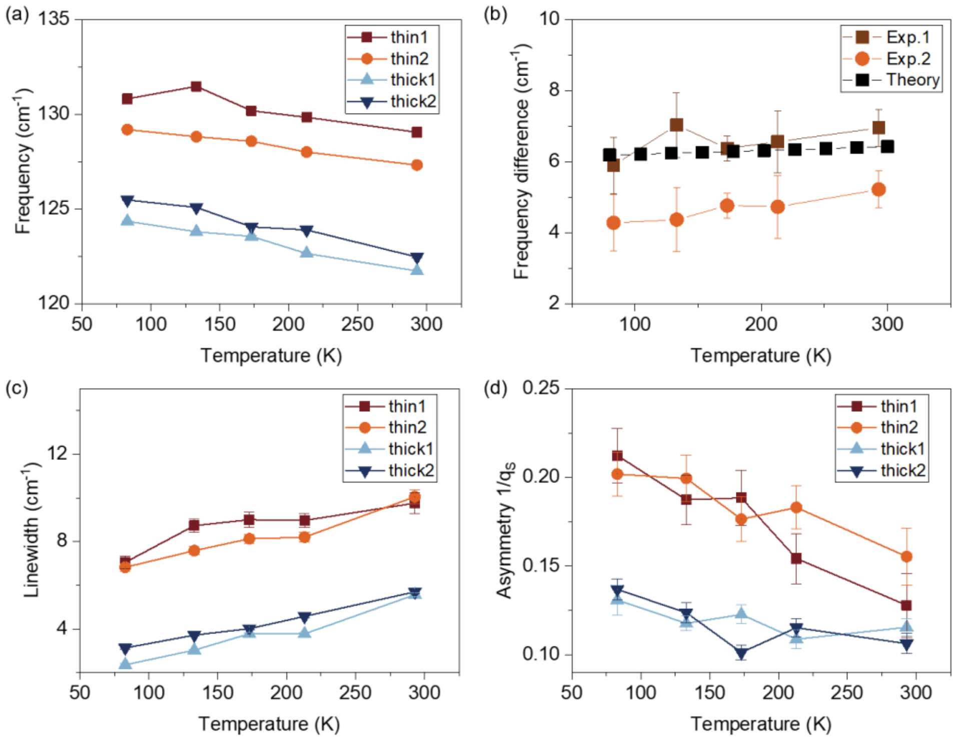

Fig. S8. Temperature dependence of the A_1 phonon mode. The four Te flakes are the same as in Fig. S7. (a) The experimental frequency as a function of temperature. (b) Frequency differences between thin and thick Te flakes as a function of temperature. Exp.1 (Exp.2): the difference between thin1 (thin2) and the average of thick1 and thick2. The error bar is the standard deviation of thick1 and thick2. Theory: theory-predicted frequency difference between small-polaron-dominated phonon and large-polaron-dominated phonon. The temperature dependence of the large-polaron-dominated phonon was taken from the fitting of the experimental thick Te. (c-d) The experimental linewidth and asymmetry as a function of temperature. The spectra are fitted with a BWF function. The error bar for the experimental data in (c-d) represents the fitting error.

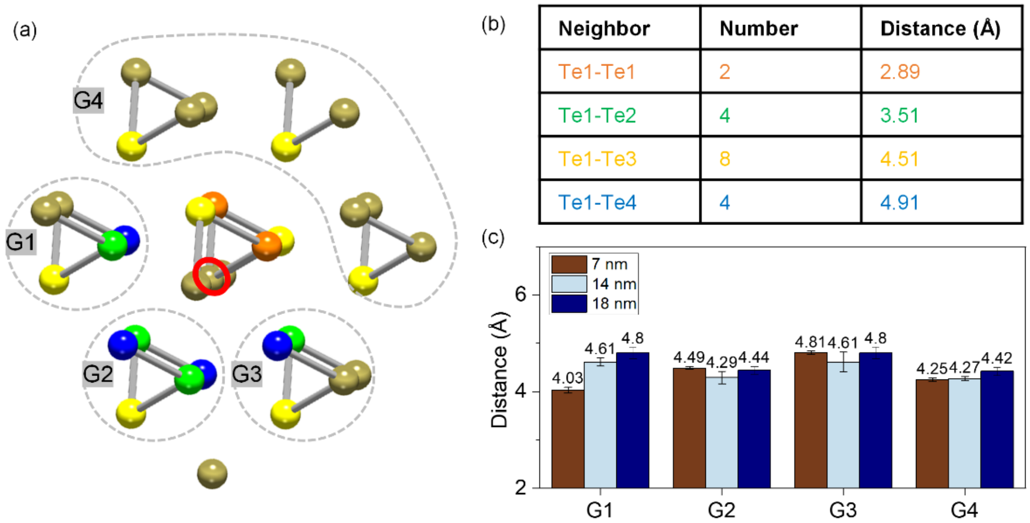

Fig. S9. Extended X-ray absorption fine structure (EXAFS) fitting model and results. (a) Illustration of the crystal structure and fitting model. The atoms colored in orange, green, yellow, and blue correspond to the atoms with a distance of 2.89, 3.51, 4.51, and 4.91 Å to the center atom circled in red. (b) The number of atoms within 5 Å to the center atom. A tellurium path of R_{eff} = 2.89 Å, N = 2 is used for the nearest atoms in the same chain, and another tellurium path of R_{eff} = 4.51 Å, N = 2 is used to account for the second nearest atom in the same chain. Fitting parameter α_i (i = 1, 2) was given for each path to describe the expansion or contraction of the nearest and second-nearest Te-Te bonds. In terms of atoms in the three neighboring chains G1-G3 that may serve as scatters, tellurium paths of R_{eff} = 3.51 Å (N = 4), 4.51 Å (N = 3), and 4.91 Å (N = 4) were assigned. For atoms in each chain, they were given the same α_i (i = 3, 4, 5 for three chains) to evaluate the interchain distance. Group G4 contains three neighboring chains with the tellurium path of R_{eff} = 4.51 Å (N = 3) and fitting parameter α_i (i = 6). The model was fitted simultaneously to the data for three different thicknesses processed by using a k-weighting of 1 and 2 in the Fourier transform. (c) Fitted interchain distance between different helical chains (each chain is composed of a group of atoms as illustrated in (a)) labeled as G1 to G4 to the center helical chain.

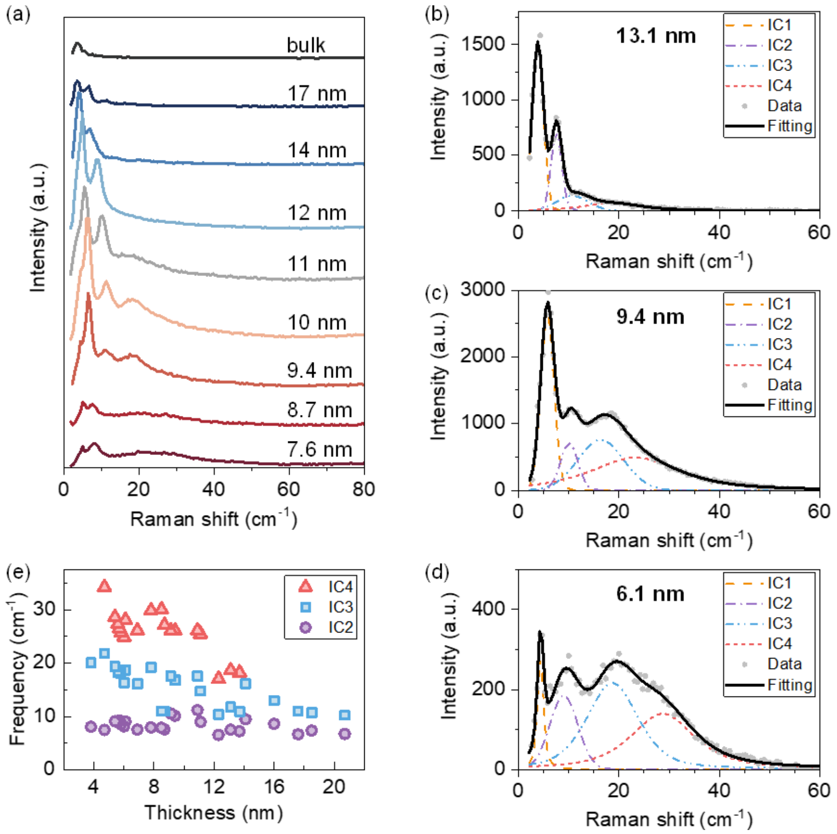

Fig. S10. Thickness-dependent interchain coupling. (a) Low-frequency Raman spectra of tellurene with different thicknesses. (b-d) Fitting of the low-frequency Raman modes for tellurene with a thickness of 13.1 nm, 9.4 nm, and 6.1 nm. (e) The Raman frequency of the interchain (IC) modes is summarized as a function of thickness. The interchain modes are labeled as IC1-IC4 based on their frequency for simplicity. The interchain mode IC1 has cut-off by the notch filter, thus, the frequency IC1 does not truly represent the phonon frequency.

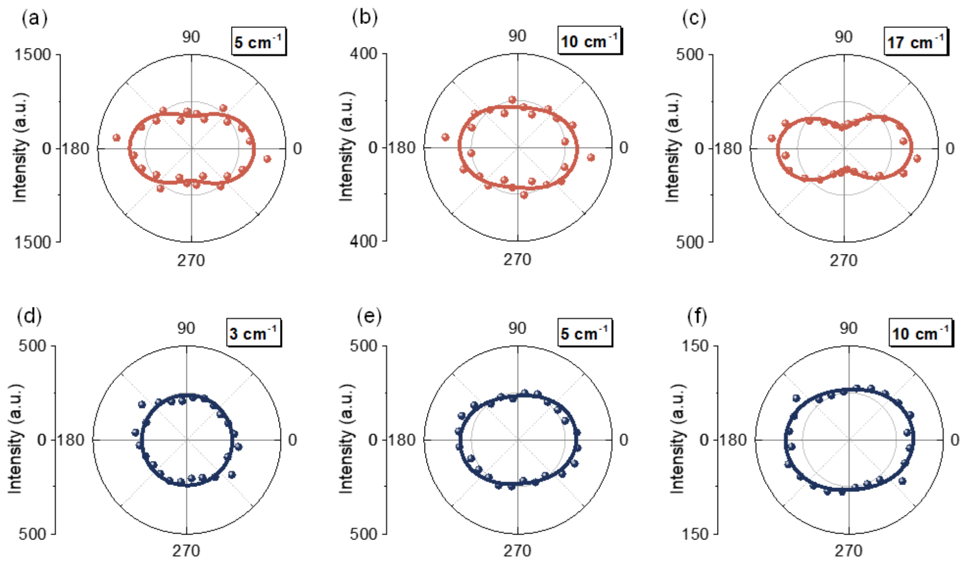

Fig. S11. Low-frequency Raman modes. Polar plots of the low-frequency Raman modes for tellurene of (a-c) 9.4 nm and (d-e) 16.9 nm. The sample was rotated in-plane and the scattering polarized parallel to the incident light was collected. The polarization angle is defined as the angle between the laser polarization and the [0001] direction.

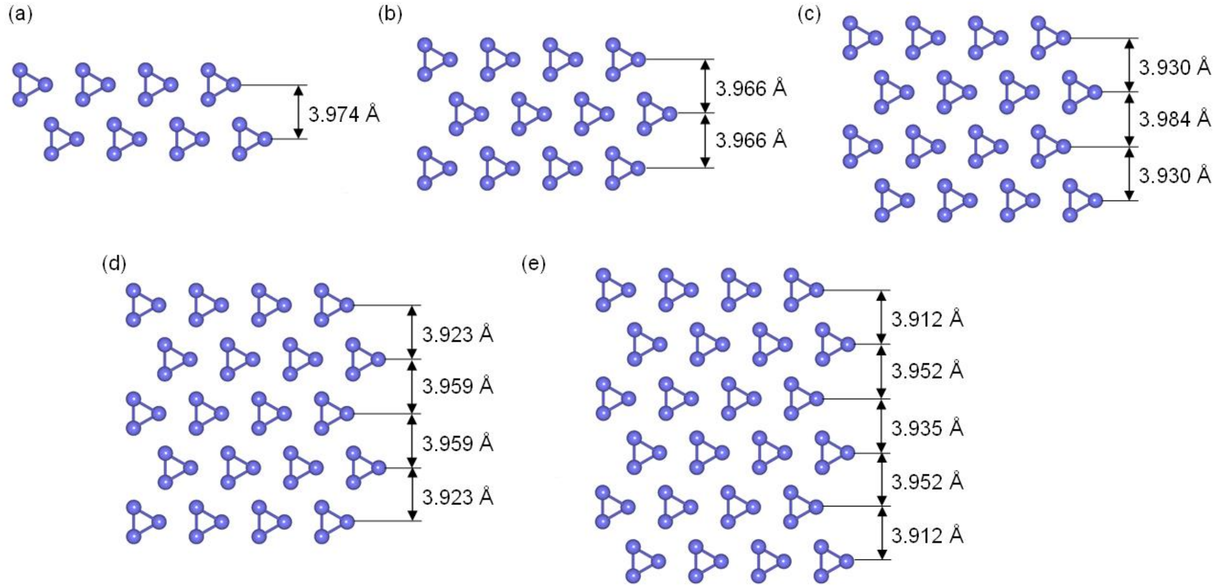

Fig. S12. The calculated vertical distance for tellurene with different numbers of layers. (a) 2L. (b) 3L. (c) 4L. (d) 5L. (e) 6L.

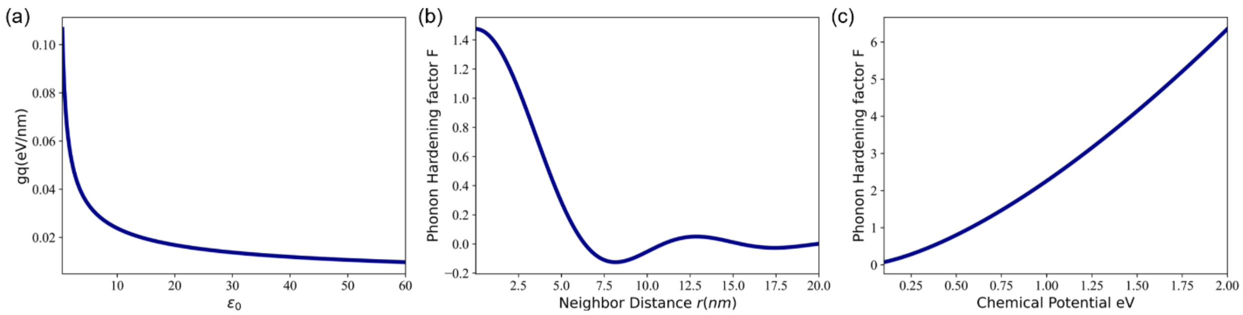

Fig. S13. Phonon properties based on polaron theory. (a) the static dielectric function dependent polar electron-phonon coupling coefficient after multiplying **q**. (b) the nearest neighbor distance-dependent phonon hardening factor. (c) the chemical potential dependent phonon hardening factor.

Table S1. Best fitting results of EXAFS. The fitting range was set as 1.85-5.00 for R-space and 3.0-6.5 for k-space. The E_0 is 6 for all fittings.

			7 nm		14 nm		18 nm	
**Scattering pair**	**Coordination number**	R_{eff}	**Debye-Waller factor**	**R**	**Debye-Waller factor**	**R**	**Debye-Waller factor**	**R**
Te1-Te1	2	2.8923	0.00414	2.8712	0.0016	2.8439	0.00473	2.877
Te1-Te3	2	4.5080	0.00598	4.2475	0.00229	4.4929	0.00683	4.7584
Te1-Te2	1	3.5069	0.00508	3.1368	0.00196	3.5840	0.0058	3.7308
Te1-Te3	1	4.5080	0.00598	4.0322	0.00229	4.6071	0.00683	4.7959
Te1-Te4	1	4.9131	0.00614	4.3946	0.00235	5.0211	0.00702	5.2268
Te1-Te2	2	3.5069	0.00508	3.4867	0.00196	3.3318	0.0058	3.4500
Te1-Te3	1	4.5080	0.00598	4.4821	0.00229	4.2829	0.00683	4.4349
Te1-Te4	2	4.9131	0.00614	4.8849	0.00235	4.6678	0.00702	4.8334
Te1-Te2	1	3.5069	0.00508	3.7337	0.00196	3.5840	0.0058	3.7308
Te1-Te3	1	4.5080	0.00598	4.7996	0.00229	4.6071	0.00683	4.7959
Te1-Te4	1	4.9131	0.00614	5.2309	0.00235	5.0211	0.00702	5.2268
Te1-Te3	3	4.5080	0.00598	4.2475	0.00229	4.2640	0.00683	4.4137
